# Born in Bradford, a cohort study of babies born in Bradford, and their parents: Protocol for the recruitment phase

**DOI:** 10.1186/1471-2458-8-327

**Published:** 2008-09-23

**Authors:** Pauline Raynor, Born in Bradford Collaborative Group

**Affiliations:** 1 Bradford Institute for Health Research, Bradford Royal Infirmary, Duckworth Lane, Bradford, BD9 6RJ, UK

## Abstract

**Background:**

Bradford, one of the most deprived cities in the United Kingdom, has a wide range of public health problems associated with socioeconomic deprivation, including an infant mortality rate almost double that for England and Wales. Infant mortality is highest for babies of Pakistani origin, who comprise almost half the babies born in Bradford. The Born in Bradford cohort study aims to examine environmental, psychological and genetic factors that impact on health and development perinatally, during childhood and subsequent adult life, and those that influence their parents' health and wellbeing. This protocol outlines methods for the recruitment phase of the study.

**Methods:**

Most Bradford women attend for antenatal care and give birth at the Bradford Royal Infirmary, which has approximately 5,800 births per year. Women are eligible for recruitment if they plan to give birth here. Babies born from March 2007 are eligible to participate, recruitment is planned to continue until 2010. Fathers of babies recruited are invited to participate.

Women are usually recruited when they attend for a routine oral glucose tolerance test at 26–28 weeks gestation. Recruitment of babies is at birth. Fathers are recruited whenever possible during the antenatal period, or soon after the birth. The aim is to recruit 10,000 women, their babies, and the babies' fathers.

At recruitment women have blood samples taken, are interviewed to complete a semi-structured questionnaire, weighed, and have height, arm circumference and triceps skinfold measured. Umbilical cord blood is collected at birth. Within two weeks of birth babies have their head, arm and abdominal circumference measured, along with subscapular and triceps skinfold thickness. Fathers self-complete a questionnaire at recruitment, have height and weight measured, and provide a saliva sample.

Participants are allocated a unique study number. NHS numbers will be used to facilitate record linkage and access to routine data. A wide range of hospital and community sources is being accessed to provide data for the women and children. Data are checked for accuracy and consistency.

**Conclusion:**

Born in Bradford will increase understanding of the factors that contribute to health and wellbeing, and identify factors that influence differences in them between people of Pakistani and European origin.

## Background

Bradford District, in West Yorkshire in the north of England, with a population of approximately half a million people, is one of the most deprived cities in the United Kingdom. It has a wide-range of public health problems that are associated with socioeconomic deprivation, including a particularly high infant mortality rate.[1] Twenty percent of Bradford city residents are of South Asian origin, almost all being from the Mirpur region of Pakistan. The population is younger than the national average, and the city also has a higher than average birth rate. Almost half the babies born in Bradford have parents of Pakistani origin (50% white; 44% Pakistani; 4% Bangladeshi; 2% other).[1] Between 1996 and 2003 infant mortality in Bradford was 9.1 deaths per 1000 live births, almost double that for England and Wales as a whole (5.3/1000). Mortality was higher for babies of Pakistani-origin (12.9/1000), than for those of European origin (7.1/1000), and worse for first generation Pakistani immigrants but improved for second and subsequent generations of immigrants. These children also have higher childhood morbidity than infants of European origin. [2-4] The city is therefore an ideal setting for research to explore the pathways linking socioeconomic deprivation with ill-health and impaired development, and for exploring differences between people of European and Pakistani origin in their experience of health, wellbeing and social capital.

The Born in Bradford cohort study will examine how genetic, nutritional, environmental and social factors impact on health and development during childhood, and subsequently in adult life. It will also examine how nutritional, environmental and social factors influence the woman's subsequent health and wellbeing. The ultimate aim will be to develop hypotheses, which can then be evaluated and tested, for health and social interventions to improve childhood and adult health.

This protocol outlines the study methods and design considerations for the recruitment phase of this longitudinal cohort study of babies born in Bradford, and their mothers and fathers. It also presents the questionnaires and other materials used for recruitment. It is anticipated that protocols for each phase of follow up of participants in the cohort will be developed. Study methods for recruitment were initially developed during 2005–6. In late 2006 through to early 2007, feasibility of the recruitment process, administration of the baseline questionnaire, and record linkage to routine data were piloted and assessed. This protocol therefore outlines the methods as agreed following the assessment of feasibility. These methods have been implemented for recruitment to the cohort since mid-2007.

### Study aims

The broad aims of the Born in Bradford cohort study are to:

• describe and compare health and ill-health within a largely bi-ethnic population

• identify modifiable causal pathways that promote wellbeing, or contribute to ill-health

• develop a model for integrating research into routine data systems within the National Health Service (NHS) in England and Wales, and potentially health care systems in other countries

• build and strengthen local research capacity

Prospective cohort studies are a resource for current and future research, potentially for many decades and across generations. Our aim is that the Born in Bradford cohort study provides a robust and broad platform for existing and future research questions. Below is a list of specific research objectives agreed as of March 2008 by the Born in Bradford Executive and Scientific Collaboration Group (see end for membership of these groups).

1. To monitor maternal and child health, and to investigate the association between genetic, obstetric, socioeconomic, ethnic and lifestyle characteristics and risk factors, and infant and childhood mortality and morbidity.

2. To determine the prevalence of congenital anomalies in Bradford, and to describe their nature and association with consanguinity and other risk factors.[5,6]

3. To compare birthweight between the offspring of South Asian and European origin mothers born within Bradford, and to determine whether the magnitude of any differences change with subsequent generations (comparing 1^st^, 2^nd ^and 3^rd^) of migrant mothers. [7-9]

4. To examine the association between birth weight and obstetric (including parity, maternal glucose tolerance, weight gain during pregnancy and complications of pregnancy) and socioeconomic factors, and family and lifestyle characteristics. Also, to explore the extent to which any of these characteristics might explain differences in birthweight between European and South Asian origin families.[10,11]

5. To determine whether the fat-thin insulin resistant phenotype seen in adults and children of South Asian origin is present at birth. That is, to determine whether, despite their lower birth weight, South Asian origin babies are more adipose (as indicated by cord leptin levels and subscapular skinfold thickness) and more insulin resistant (as indicated by cord insulin and lipid levels) than European origin babies.[12,13]

6. To examine the extent to which women's obesity, weight gain during pregnancy, modifiable behaviours and gestational diabetes mellitus contribute to insulin resistance, diabetes, and obesity in their offspring during infancy and early childhood, and to determine whether any of these effects differ between South Asian and European origin ethnic groups. [14-16]

7. To investigate differences in health, and in social and cultural environment and experiences, between people of South Asian and European origin, and to explore how any such differences might influence outcome at birth and postnatal health for both the woman and child. [17-20]

8. To investigate if there are ethnic differences in renal anthropometric indices and explore if there is an association between birth weight and renal size. Also to determine which parental and pregnancy related factors might explain such differences. [21-23]

9. To investigate the association between gestational diabetes mellitus and adverse obstetric outcomes.[24,25]

10. To examine the association of maternal vitamin D levels with fasting and postload glucose levels and with maternal blood pressure, and to examine the association of maternal vitamin D levels with offspring birth size, infant size, cord leptin, insulin and lipids, and future markers of cardiovascular risk factors in infancy and childhood.[26,27]

11. To investigate the association between dietary exposure to chemicals with carcinogenic and immunotoxic properties, intermediate markers of cancer risk and immune disorders. [28]

12. To investigate the relationship between occupational factors, traffic-related air pollution and chlorination disinfection by-products in drinking water and intrauterine growth restriction.[29,30]

13. To describe the growth and development of children in a bi-ethnic cohort, and to explore the nutritional and environmental factors that influence growth and development during infancy and early childhood.[11,31]

14. To identify symptoms of emotional and physical distress in pregnancy, and explore the meanings given to them by women of different cultural and ethnic backgrounds. [32-34]

15. To describe the epidemiology of infant and early childhood infections, and explore the hypothesis that viral infections in early life influence the risk of later immune-related diseases.[35,36]

## Methods

Bradford is served by a single maternity unit, at the Bradford Royal Infirmary (BRI), with approximately 5,800 deliveries per year. Almost all women resident in Bradford book, and give birth, in the maternity unit. Only about 10 women per year have a home birth, and a small number are transferred *in utero *to another unit, or deliver elsewhere for other reasons. The eligible population for this cohort study is all pregnant women who plan to, and then do, give birth at the BRI over the period of recruitment, their subsequent live born offspring, and their partners. Over time 'new' parental figures for the offspring will become part of the eligible study population.

### Eligibility and exclusion criteria

#### For the women

All women planning to give birth at the BRI will be eligible for recruitment, regardless of parity. Women who have been recruited to the study will remain in the study, if they wish to, regardless of whether the baby is liveborn or stillborn, and irrespective of whether the child is adopted or fostered.

The only exclusion criterion for recruitment of a woman will be if she plans to move away from Bradford before the birth. As recruitment to the study is anticipated to take place over 36 months, some women will have more than one pregnancy during this period. The woman, and her subsequent offspring, will be eligible for each pregnancy.

#### For the children

All babies born to women who have agreed to participate in the cohort study will be eligible for recruitment. If the woman does not want to participate in the cohort herself, she can still agree that her child participate.

For stillborn babies recruitment to the cohort will be at birth, as it is for liveborn babies. For these babies there would, of course, be no further participation.

#### For the fathers

Fathers of babies who have been recruited to the cohort will be invited to participate when the mother attends for her oral glucose tolerance test, at another convenient time during pregnancy, or soon after the birth. The normal procedure will be to invite the woman's current partner to participate, regardless of whether or not he is the baby's genetic father. If there is uncertainty about who to approach, the woman will be asked who should be offered participation. If the woman has a new partner in the future, he will also be invited to participate.

#### For adoptive parents and foster carers

Adoptive parents and foster carers will be invited to participate in the study, if appropriate.

### Recruitment

The study aims to recruit 10,000 babies. Recruitment began in March 2007 and is planned to continue until 2010. Approximately half the babies born in Bradford have parents of South Asian origin, primarily from Pakistan. People from ethnic minorities are as likely as those from majority populations to agree to participate in research, provided they receive an appropriate invitation in a language they understand.[37,38] We will do our utmost to ensure that the study is appropriately explained and that language is not a barrier to participation. We therefore estimate 80% uptake for the women and children, with 75% retention over five years. Uptake and retention for the partners is likely to be considerably lower.

#### Ethnicity

Mothers are asked to self report their own and the baby's father's ethnicity, where they were born and how long they have lived in this country. They are also asked to state the ethnicity of theirs and the baby's father's parents and grandparents. If fathers are recruited into the study they are requested to self report to which ethnic group they belong, where they were born (UK, Pakistan or other) and how long they have lived in this country.

Information about the study has been widely disseminated throughout the community, in appropriate languages, using a range of media such as local newspapers, video, radio and TV and the internet through a project website. Links have been developed with community organisations, including the primary health care trust, mosques, other faith groups, schools and colleges. Written information about the study has been made widely available, so that families are aware of the study before they attend for antenatal care.

Women are given the Patient Information leaflet (see Additional file 1) at their first antenatal appointment. They have an opportunity to ask questions and discuss the project at subsequent antenatal visits. This leaflet is also available to women when they come to the BRI for antenatal appointments and for ultrasound scans. Muslim women are offered an additional leaflet that discusses research and the Born in Bradford project from an Islamic perspective (see Additional file 2).

Most people of Pakistani origin living in Bradford speak either Urdu or Mirpuri. The Patient Information leaflet has therefore been translated into Urdu. As Mirpuri does not have a written script, the patient information was transliterated (see 3.5 below). The other non-English speaking communities represent less than 3% of deliveries, and are largely from Eastern European countries such as Poland, Slovakia and the Czech Republic. These women are recruited with the aid of interpreters, whenever possible, and a summary leaflet outlining the recruitment process and inviting participation has been translated into Polish, Czech and Slovakian (see Additional file 3).

### Sample size

Comparable cohorts to estimate feasibility of recruitment are scarce. ALSPAC is a slightly larger cohort study which recruited more than 10 years ago. Women were recruited earlier in pregnancy than proposed for our study, and less than 10% were 'non-white'. Recruitment to ALSPAC was estimated to be 80–90% of eligible women, and 551 were excluded because they miscarried before 20 weeks gestation. Of the 13990 women who delivered after 20 weeks, 69 (0.5%) were lost to follow up before delivery. Among the 14130 babies born after 20 weeks, 68 were stillborn and 53 were neonatal deaths. Response rates to postal questionnaires were around 80% at five years, for both women and children. For the fathers they were around 50%.

Although our study will have a much larger proportion of women and partners from ethnic minorities, there is a growing body of evidence that people from ethnic minorities are as likely as those from majority populations to agree to participate in research. The main barriers to participation are that an invitation is not offered, or that it is in a language the person does not understand. Once people are invited to participate in a language they understand, the likelihood they will accept does not seem to be related to their ethnic group. The challenge for the Born in Bradford study will therefore be to ensure that language is not a barrier to full participation. We estimate 80% uptake for the women and children, with 75% retention over five years. Uptake and retention for the partners is likely to be lower, at 50% uptake and 70% retention.

#### Recruitment of the women

All women booked for delivery at the BRI are offered an oral glucose tolerance test (oral GTT) at 26 to 28 weeks gestation. When they attend for the oral GTT, women are invited to participate in the Born in Bradford cohort. If they agree, they are asked to first give verbal consent to having a project blood sample (total 13 ml) for inclusion in the Biobank, taken at the same time as the fasting oral GTT sample. Once these blood samples have been taken, women have the opportunity to discuss the study in detail with a member of the project team, and to decide if they wish to sign a consent form (see Additional file 4). Once this consent form has been signed, women are invited to complete the baseline questionnaire with a trained project worker (see Additional file 5), and to have anthropometric measurements taken. Most of the questionnaire is completed by interview with the project worker, with the final section completed by the woman herself (see below).

After having blood taken, if the woman decides she does not wish to participate she can request that her samples be destroyed. If verbal consent to take extra blood is declined, the woman can still be recruited to the study. Similarly, if a woman has already had the fasting blood sample taken for her oral GTT before she is approached to ask about participation in the cohort, she can nevertheless be invited to participate.

Overall, 80% of women who are booked to give birth at the BRI attend for the oral GTT. Women who do not attend or who, for operational reasons, are not approached during their visit for the oral GTT, are invited to participate at other times. For example, women with pre-existing diabetes are invited to participate when they are at 28 weeks gestation, or above, during a clinic appointment, or at another appropriate opportunity. Women who have not been offered participation are also invited to participate when they attend the pre-assessment clinic. If a woman has been invited to participate and refused, she will not be approached again.

#### Recruitment of the babies

Women who sign the consent form thereby give consent for their babies to be included in the study. These babies are then recruited into the cohort at birth, regardless of whether they are liveborn or stillborn.

#### Recruitment of the fathers

Fathers who accompany women when they attend for the oral GTT are also invited to participate. If they agree to take part and sign the consent form (see Additional file 6), they have height and weight recorded, and are asked to complete a self administered questionnaire (see Additional file 7) and to provide a saliva sample. If the father was not approached during the oral GTT visit, he is invited to participate if he accompanies his partner during other hospital visits, or after the birth.

#### Inclusion of women and fathers following a stillbirth, neonatal death or infant death

Following a stillbirth, neonatal death, or infant death the women and fathers will remain in the cohort. Future follow up will ensure that any contact with the family is sympathetic and appropriate.

#### Withdrawal of consent

A woman may withdraw from the study, or withdraw her child, at any time. Once a request for withdrawal has been received a letter is sent to the mother thanking her for her participation and confirming if she wishes any data collected for her and/or her child up to that point in time (biological, questionnaire or routinely collected information) to be destroyed. Similarly, a father may withdraw from the study at any time. They will not be contacted again, and no further data will be collected for her and/or her child. Data and biological samples that have already been collected for a participant who later withdraws will be retained for analysis, unless consent for storage and analysis has also been withdrawn, in which case they will be destroyed.

### Unique study identifier

Each woman is allocated a unique study number at recruitment. This number will be used to identify and track her anonymised data throughout the study. It is vital for linking information across the study, and for ensuring confidentiality. Similarly, the woman's child and her partner are allocated a unique identifier, but one that is linked to hers. Hence, each family unit will be identified and linked, whilst maintaining confidentiality. The system for allocating study identifiers also allows a new partner to be added to the family unit, and identifies second and subsequent pregnancies during which the woman is recruited to the cohort.

In addition, NHS numbers are collected for all study participants. These will be used to facilitate record linkage. Demographic details collected when the consent form is signed are validated via the National Strategic Tracing Service (NSTS), soon to be replaced by the Patient Demographic Service (PDS), to check the NHS number and identify the general practitioner.

### Translation and transliteration of trial materials

Most people in Bradford who describe themselves as Pakistani come from families who migrated from the Mirpur region of north eastern Pakistan. Although there are some smaller Pakistani communities, the vast majority of people are able to understand Urdu or Mirpuri. Literacy in these languages is not universal, however. A further complication is that Mirpuri does not have a written form in use; it is sometimes regarded as a dialect of Punjabi rather than a separate language. To reduce the need for translators, and to facilitate consistent and accurate data collection, the baseline questionnaire has been transliterated into Urdu and Mirpuri using a standardised process (see Additional file 8). Bilingual project workers who administer the questionnaire have been trained in use of the transliterated versions.

### Routine data to be collected at recruitment, and up to the time of the birth

The aim is to maximise the utility of routinely collected data within the NHS systems, hence potentially providing comprehensive and cost effective data collection. A wide range of sources will be accessed to provide baseline data for the women and children at recruitment; for example haematology and biochemistry results, ultrasound data, and general practice data.

#### For the mother

Information about the women recorded as part of routine clinical care at booking and throughout pregnancy and at delivery will be obtained from hospital information systems (see Additional file 9). A new maternity electronic record (eClipse) is being installed at the BRI; designed to be completed by the care givers and used as the primary clinical notes from booking to postnatal discharge. The aim is that this will be a 'paper light' system, with the advantage of clinical data being routinely collected in electronic format, and therefore readily available for clinical care whenever needed. An additional advantage is that, with appropriate ethics approval and consent, it can also be accessed for research purposes. While the transfer from traditional records to eClipse is taking place, data that are not available electronically are being manually extracted from the paper records.

#### For the baby

Information about the baby at birth is also being collected within eClipse as part of routine care, and is therefore available from these electronic records.

#### For the father

No routinely collected data will be accessed either at recruitment or around the time of the birth for the father.

### Project data to be collected at recruitment, and up to the time of the birth

As routine data may not provide comprehensive and reliable information about issues such as ethnicity, culture, well being, lifestyle, and mental state, supplementary data for the women and fathers will be collected using semi structured questionnaires (see Additional files 5 and 7). All women and fathers will be offered the opportunity to complete these questionnaires. However, if the questionnaire is not completed, for whatever reason, they can still participate in the study if they wish.

#### For the women

Women will be weighed and have height, arm circumference and triceps skinfold measured at recruitment. The baseline questionnaire developed for this study (see Additional file 5) will be administered by a trained project worker at recruitment, regardless of whether that occurs during the visit for the oral GTT or at another time. The questionnaire is designed to be completed as part of a semi-structured interview, with a final section containing the General Health Questionnaire 28 (GHQ 28) and exercise questions being self completed. The questionnaire includes already existing and previously validated questionnaires where ever possible, plus additional questions developed by the Born in Bradford project team for topics on which appropriate existing questions could not be found. Its content reflects baseline data needs defined by the broad aims and specific objectives of Born in Bradford (see above). The source for each question in the questionnaire is listed in Additional file 10.

The project worker administering the baseline questionnaire will remain with the woman while she completes the self-completed section, and will be willing to answer questions. This same project worker will check the self-completed section has been fully completed before the woman leaves. If the woman indicates either by her answers to the final section of the GHQ 28, or in conversation, that she has suicidal thoughts or feelings, there will be a standard procedure for informing the woman of counselling and other support that is available to her.

In addition to the baseline questionnaire, a dietary questionnaire will be given to all women recruited to the study while they are waiting for their oral GTT. They will be invited to complete this questionnaire and return it to a member of the project team before they leave. This questionnaire will be self-explanatory, and will be completed without the assistance of the project workers.

#### For the babies

At, or soon after, birth the babies will have their head, arm and abdominal circumference measured, along with subscapular and triceps skinfold thickness.

#### For the fathers

The fathers' baseline questionnaire (see Additional file 7) is also administered at recruitment. This questionnaire is self completed. Fathers have their height and weight measured.

### Biological samples to be collected and stored at recruitment

As outlined above, assay results from biological samples collected and analysed within the context of routine care for the women and babies will be available for those who have consented to recruitment.

#### For the women

Women who are recruited at their oral GTT have an additional fasting blood sample taken for the project. This is used to measure insulin, glucose, and lipid profile, with 13 mls then prepared for storage at -80°C: serum, whole blood, plasma, buffy coat and red blood cells. A single void urine sample of approximately 50 ml will be collected into sterile specimen containers, and stored at -80°C in the Biobank.

#### For the babies

At birth, umbilical cord blood will be collected for the Biobank using the Vacutainer system The following samples will be stored at -80°C: serum, whole blood, plasma, buffy coat and red blood cells.

#### For the fathers

Fathers will be asked to provide a single saliva sample at a convenient time (such as when accompanying their partner to the oral GTT), for storage at room temperature in an Oragene^® ^(DNA Genotek, Ottawa, Ontario, Canada) specimen container. This will be used as a source of genomic DNA.

### Data quality and data management

A range of strategies will be utilised to maximize the quality of data collection. This will include validation checks for consistency and completeness of routinely collected data, training of project workers in administration of the questionnaires, and training of clinical and project staff in measurement of specific indices.

#### Training and quality assessment of project workers in administration of the women's questionnaire

All project workers who recruit to the study and administer the baseline questionnaire have initial training in how to explain the study, how to take consent and how to administer the questionnaire using a range of strategies, such as formal training sessions, practical demonstrations and role play. A Born in Bradford training manual is used to ensure standardization of instructions and consistency of training standards for project workers throughout the life of the project. Once they begin recruitment, each project worker has a random sample (5%) of interviews observed by an independent observer on a quarterly basis. A set of interviewer notes is also provided to help standardise explanation of specific issues, and to eliminate ambiguity (see Additional file 11).

#### Training and quality assessment for anthropometric measurements

Everyone who takes anthropometric measurements, which includes both project workers and clinicians, is trained in the appropriate technique. Training includes how to check, calibrate and maintain the necessary equipment.

The Technical Error of Measurement (TEM) is used to assess reliability of the anthropometric measurements. TEM is calculated from the data produced by a test-retest reliability study (see Additional file 12). This requires the measurer to assess the same infant twice (to assess intra-observer reliability) or two or more observers to assess the same infant (to assess inter-observer reliability). Until there are sufficient data to assess reliability within this study, the acceptable ranges of difference between two measurements on the same person have been agreed (see Additional file 13).

### Data warehouse

**Figure 1:**
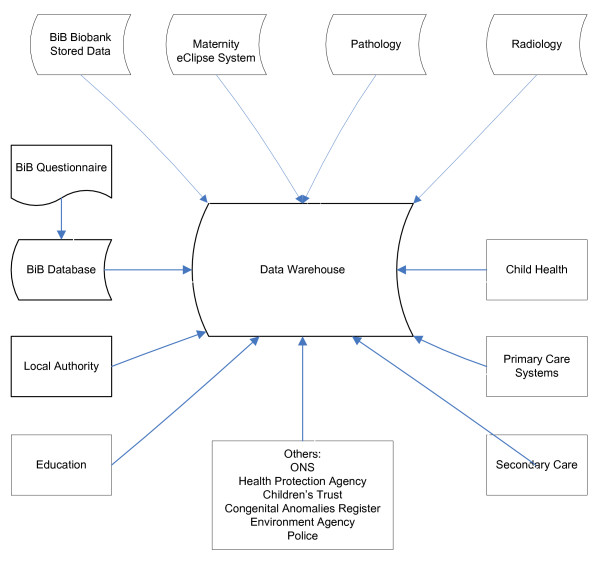
Sources of data from the Born in Bradford Data Warehouse.

All study data will be linked using NHS numbers and the unique study identifier, and stored in a data warehouse. This includes data from the clinical analysis of blood samples, data from the baseline questionnaires, and routine clinical data (Figure 1). Data linkage, quality control, and maintenance of the data warehouse are the responsibility of Bradford and Airedale Teaching Primary Care Trust, on behalf of Bradford Hospitals NHS Foundation Trust.

#### Data consistency

If a data item is available from more than one source, an agreed process is used to validate the data and create a single field containing the most reliable information. For example, if ethnicity is collected from four sources, the most common response would be used.

Where similar data are collected from more than one source these are clearly documented. Information will be provided to users of the data to enable them to choose the field most appropriate to their requirements, or to consider combining information from more than one field when feasible or appropriate. All processes for data management and quality control are clearly documented and agreed by the Data Management Group.

#### Data linkage

NHS numbers will be used to enable regular agreed extracts of routinely collected data. Some systems, such as those for maternity, child health and primary care, may already have Born in Bradford study participants identified within them. Nevertheless, these will be regularly cross-checked before data extraction to ensure any changes in consent have been taken into account. Data obtained in this way will be cross checked for consistency, plausibility, and completeness. Further checks will validate data across the different sources. Discrepancies and inaccuracies will be checked and investigated.

Systems for data linkage to education and social services records are being developed.

### Storage and tracking of samples for the Biological Bank

A number of principles have been agreed with respect to storage of the biological samples. First, samples should be processed in such a way as to enable the widest possible range of analytical tests to be conducted. This is in recognition of the fact that it is not possible to predict from the outset the scope of specific analyses. Second, the protocol seeks a balance between the ideal intensity of sampling for scientific purposes and the practical and logistic setting in which the work is conducted. Third, samples are to be processed in a timely fashion and there should be an avoidance of freeze-thaw cycles. Fourth, cataloguing and storage of samples will be subject to a secure and robust inventory system. Fifth, best practice will be followed to maintain the highest standard in clinical and research governance, including fulfilling our obligations and responsibilities laid down in the ethical and human tissue authority legislation.

Once the samples have been collected, according to the procedures detailed above, the Biobank has a system for recording the exact location of each sample and its storage conditions. All samples are collected and processed following agreed Standard Operating Procedures (SOPs). All deviations from these SOPs, such as temperature changes or freeze/thaw episodes, and equipment failures, are recorded. Review of all storage equipment will be done annually. For each specimen the time from collection to storage is recorded. All aliquots and samples have a unique code to allow sample tracking. Consent for samples to be stored, and indeed where consent has been revoked, is tracked so that if consent is withdrawn samples can be destroyed in a timely manor, and this procedure recorded in the study database. A procedure will be put in place for disposing of samples when consent is withdrawn, or when a sample has not been collected in an acceptable time-frame.

### Research governance and confidentiality

Research Governance approval has been secured from Bradford Teaching Hospitals NHS Foundation Trust and the Bradford and Airedale Teaching Primary Care Trust. Data collection will comply with the Data Protection Act 1998. Files containing personal identifiers (such as name, address, telephone number) will be stored in locked cabinets or rooms within the project co-ordinating centre, and separate from the data used for analysis. Researchers who are not part of the project co-ordinating centre (including those on the Executive and Scientific Collaborators groups) will not have access to personal identifiers. Back up copies will be subject to the same degree of data security. Data will be released for analysis using the unique study identifier only, hence protecting the identity of individual participants.

### Ethics approval and indemnity

The protocol for recruitment and collection of baseline data and biological sample for the cohort has been approved by the Bradford Research Ethics Committee. All participants will be asked to sign a consent form (see Additional files 4 and 6), which includes permission to use genetic data obtained from biological samples. Standard procedures for the protection of confidential individual information will be applied, as outlined above. Samples will be collected and stored in accordance with the Human Tissue Act 2004, including ensuring appropriate licences are in place.

### Analysis plan

Throughout the initial data collection period regular comparisons will be made between women recruited to the study, and those eligible but not recruited, using routinely available data for women who book to give birth at the BRI. These comparisons will be used to target recruitment for particular groups, as appropriate. For example an initial analysis of the first 500 women recruited to the cohort demonstrates that our cohort is highly representative of the eligible population with one exception: fewer very young (under 18 years) pregnant women were recruited compared to those in the eligible population. As a result, community midwives and ante natal support services working with this age group have been asked to discuss the study with young pregnant women. Representativeness of this cohort will continue to be monitored throughout data collection, with appropriate strategies developed to rebalance recruitment should any group be found to be under-represented. A final report on characteristics and demographics for the whole cohort will be produced at the end of recruitment.

For each project planning to use the Born in Bradford cohort, an analysis plan will be developed before commencing data analysis.

### Project management

Successful conduct of this study will require considerable input from a wide range of people locally within the Bradford Teaching Hospitals NHS Foundation Trust, University of Bradford, Bradford and Airedale Teaching Primary Care Trust, University of Leeds and academic partners from collaborating institutions: University of Bristol, University of Edinburgh, University of Loughborough, London School of Hygiene and Tropical Medicine, Imperial College London.

#### Executive Group

This group has responsibility for oversight of the project, including: monitoring project progress against milestones, oversight of the study conduct, strategic planning, planning grant applications, co-ordinating nested study applications, oversight of the budget, ensuring the project meets the requirements of Research Governance and data protection. The Executive Group are also responsible for producing quarterly reports for the Trust Board.

#### Project Management Group

This group meets every one to two months and is responsible for day to day implementation of the project, including co-ordination of clinical and research staff contributing to the project, development of the supporting IT systems, training and awareness of clinical and research staff, monitoring of monthly recruitment, and monitoring of collection, analysis and storage of biological samples.

#### Scientific Collaboration Group

This group meets quarterly and provides academic review and support for the project. Specific roles include: contributing to the design and methods of the project, collaboration on research proposals for funding, advising on dissemination of research results, providing strategic scientific direction.

#### Advocacy and Scrutiny Committee

This committee acts as an independent body and has a broad remit to review and comment on the overall direction and intentions of Born in Bradford as well as specific areas, including new questions; new measures including biological samples; nested studies; protocol based prospective measures and participant information. Its structure, membership and operating procedures are in Additional file 14. The group reports to the Trust Board via the Executive Group.

### Intellectual property and principles of collaboration

Born in Bradford is a platform study that aims to increase our understanding about how we can improve health and well-being within Bradford, and to contribute to our understanding of the causes of disease and promotion of health more widely. It also aims to increase research capacity in Bradford by offering opportunities for local researchers, and by attracting the involvement of high calibre external research partners. Implementation of the study is dependent on the commitment and contributions of a wide variety of health professionals and researchers, including midwives, obstetricians, paediatricians, laboratory staff, health visitors, health service managers and general practitioners. But it also depends on active participation by the parents and children recruited to the study, and the many community groups and individuals who support that involvement. The project policy on governance, intellectual property and authorship is in Additional file 15.

Born in Bradford is therefore dependent on the input and contributions of a large number of stakeholders. This plurality is a strength of the study, but may also lead to confusion over ownership. All research outputs from the study should clearly acknowledge the contribution from the Bradford community. The cohort study has been set up to attempt genuine public engagement and the Born in Bradford cohort should therefore be seen as active contributors rather than just passive participants.

Proposals for collaboration or for projects that use data from the cohort from external groups are welcome, but until recruitment is completed there cannot be any guarantees about data availability. Once all data have been collected and validity and consistency checks completed, the study will be promoted more widely. For example, by convening a preliminary meeting for potential external researchers at which the cohort will be described, along with presentation of some initial descriptive data. At that stage proposals for future research collaboration will be actively encouraged.

Researchers with proposals to use data from the cohort should in the first instance complete an outline pro forma available on the Born in Bradford website.[39] These will be reviewed by the Executive group. Those agreed by the Executive Group will be discussed with the Scientific Collaborators Group. They will also be made known to the Advocacy and Scrutiny Committee who will be invited to comment. The principles for agreeing a proposal and releasing data from the study to external researchers are that the proposal (a) meets the broad study aims; (b) outlines research that has not already been completed, is in progress or has been agreed with another group of researchers; (c) is feasible, and realistically budgeted, in the Born in Bradford cohort; (d) will not bring the study into disrepute or cause offence to the participants. Where appropriate, collaboration with Bradford researchers will be particularly encouraged, in order to build local research capacity.

## Competing interests

The authors declare that they have no competing interests.

## Authors' contributions

This protocol was prepared by members of the Executive Group, with input from members of the Scientific Collaborative Group. The draft has been seen and agreed by members of the Born in Bradford Collaborative Group.

## Pre-publication history

The pre-publication history for this paper can be accessed here:



## Supplementary Material

Additional file 1 Parents' information sheet. Information leaflet sent out to families before they are approached to take part in Born in Bradford. Click here for file

Additional file 2 Islamic Perspective Leaflet. Leaflet offered to Muslim women outlining the aims of the project from an Islamic perspective. Click here for file

Additional file 3 Polish, Slovakian and Czech Leaflet. Summary leaflet handed out to Polish, Slovakian and Czech mothers explaining the study and offering the help of an interpreter if they wish to take part. Click here for file

Additional file 4 Mothers' Consent Form. Mothers' Consent Form. Click here for file

Additional file 5 Mother's Questionnaire. Baseline questionnaire completed by mothers who agree to take part in Born in Bradford. Click here for file

Additional file 6 Fathers' Consent Form. Fathers' Consent Form. Click here for file

Additional file 7 Father's Questionnaire. Self-completion questionnaire given out to fathers who wish to take part in Born in Bradford. Click here for file

Additional file 8 Translation and transliteration process for Urdu and Mirpuri versions of questionnaire. Flowchart outlining process involved in developing the Urdu and Mirpuri transliterated versions of the baseline questionnaire. Click here for file

Additional file 9 Routinely collected data. List of routinely collected maternity data to be extracted from eCLipse the Maternity IT system. Click here for file

Additional file 10 Origin of questions. List of the origins of each question included in the questionnaire. Click here for file

Additional file 11 Interviewer notes. Notes provided to those administering the baseline questionnaire to standardise the way the questions are asked. Click here for file

Additional file 12 Quality control standards for growth measurements. Quality monitoring standards for collection of anthropometric measurements. Click here for file

Additional file 13 Table 1. Acceptable ranges of difference between the two measurements on the same person. Click here for file

Additional file 14 Advocacy and scrutiny committee. Terms of reference for the Advocacy and Scrutiny Committee. Click here for file

Additional file 15 Guidance and conditions for collaborators on the Born in Bradford programme. This document outlines the procedures put in place to deal with applications to conduct nested studies; requests to access and use study data and publication and intellectual property protocols. Click here for file
