# Supplementary material for: Born in Bradford, a cohort study of babies born in Bradford, and their parents: Protocol for the recruitment phase
Source: BMC Public Health. 2008 Sep 23;8:327. doi: 10.1186/1471-2458-8-327 (PMC2562385; doi:10.1186/1471-2458-8-327)
Supplement: Additional file 1 — Parents' information sheet. Information leaflet sent out to families before they are approached to take part in Born in Bradford. [file 1471-2458-8-327-S1.pdf]

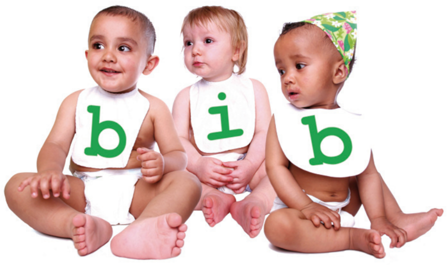

Bradford Teaching Hospitals NHS
NHS Foundation Trust

Parents' Information Sheet

Please read this information sheet carefully

This information sheet gives details of a research project set up at Bradford Royal Infirmary to study the health and well being of children and their parents. The project is called Born in Bradford. Please take some time to read the following information carefully.

- **Why are we carrying out Born in Bradford?**

The aim of the project is to study the health and development of children from birth and as they grow up. The project will also study the health of mothers and fathers. All the information we collect will help us understand the causes of illness, which will help us to develop better ways of improving health and well being.
We are inviting every pregnant woman coming to the Bradford Royal Infirmary antenatal clinic to take part in the Born in Bradford project.

- **What will happen to me if I take part?**

Whether you decide to take part in the project is entirely up to you. If you decide you want to and later you change your mind, you can withdraw. Whatever you decide your medical care will not be affected in any way. If you decide to join the project, we will tell your GP that you are taking part.
As part of the research you will be asked to

- Sign a consent form
- Complete a questionnaire with the help of someone from the project, which takes approximately 30 minutes, this is done during your routine Glucose Tolerance Test appointment
- Donate a small amount of extra blood which will be taken when you have a routine blood test
- Complete further short questionnaires about your baby as they grow.

- **What will happen to my child if I take part?**
  - After the cord has been cut and you and your baby have separated from the cord and placenta, we will take a small amount of blood from the umbilical cord. This will not hurt your baby.
  - Take a sample of your baby's first stool/bowel movement, called meconium.
  - Record growth measurements, immunization and illness event

- **Fathers**

Fathers will be offered an invitation to join the study around the time your child is born. If they accept, fathers will be asked to complete a short questionnaire and they will be asked to give a saliva sample. If the father of your child decides not to accept the invitation, you and your baby can still participate in the project.

- **What will happen to the information we collect?**

Information will be stored for use by researchers from the Born in Bradford project and their research partners in the UK and both inside and outside the European Economic Area. Some of these countries do not have the same data protection laws as in the UK, however the information will only be labelled with a study number, so it cannot be linked to you.

- **What will happen to the samples we collect?**

Samples collected such as blood, urine and saliva will be stored and kept for use by the Born in Bradford project and their research partners in the UK and both inside and outside the European Economic Area. Samples will be used to investigate the causes of a variety of diseases such as diabetes, heart disease and childhood growth. We will also look for genetic features and factors related to where you work or live which may contribute to the development of diseases in later life. No individual results will be available

- **Keeping in touch**

An important part of Born in Bradford is to keep in touch with those who have agreed to take part. To help us do this, we will use information held by the National Health Service (NHS) and by the Office of National Statistics (ONS) (this is a national organisation which provides health information for people living in England and Wales). If at any time you decide you do not want us to contact you again, you just have to tell us.

- **How do we ensure confidentiality?**

All the information we collect about you, and the samples we take from you, will be stored in strict confidence, as is required by law in the Data Protection and Human Tissue Acts. A study number will be the only way the information can be linked to you. The information for Born in Bradford will be stored in a large computer. Only certain members of the project team will be able to link you to the information we have collected about you. This is important so that we can contact you in the future.

- **Are there any possible advantages of taking part in the project?**

Some women say they enjoy taking part in research that may help other women and their babies in the future. Others find talking to a researcher about themselves and their pregnancy can be helpful. The main advantage of taking part is that you will be helping us in our efforts to understand more about the health of people living in Bradford, and how this might be improved for future generations.

- **What if I have any questions or problems?**

A researcher will talk through the project with you when you attend the hospital. You can also contact us at the Project Office on 01274 364474 and speak to someone from the team. If you have any questions about your general health or pregnancy, you should contact your doctor, midwife or health visitor.

- **Will I get to know the findings of the project?**

The findings from the Born in Bradford project will be published in scientific journals, but we will also make sure they are reported in newspapers, and on the TV and radio. To make sure people in the study have the opportunity to hear about the findings, we will hold public meetings, send results to community groups in Bradford, and to GPs. None of these publications or presentations will identify individual people.

Thank you for reading this Information sheet and considering taking part in this study

The Bradford Research Ethics Committee, and the Bradford Teaching Hospitals research and development department have approved the project.

You will be approached by a member of the research team or your midwife with an invitation to take part in the project. If you do not wish to be approached would you please let a member of staff know at the maternity reception desk when you attend for your first hospital appointment.

Contact Numbers:
Project Office – 01274 364474
Web site: www.borninbradford.nhs.uk
