# Supplementary material for: Born in Bradford, a cohort study of babies born in Bradford, and their parents: Protocol for the recruitment phase
Source: BMC Public Health. 2008 Sep 23;8:327. doi: 10.1186/1471-2458-8-327 (PMC2562385; doi:10.1186/1471-2458-8-327)
Supplement: Additional file 2 — Islamic Perspective Leaflet. Leaflet offered to Muslim women outlining the aims of the project from an Islamic perspective. [file 1471-2458-8-327-S2.pdf]

Leaflet Inner.1 21/9/06 15:51 Page 1

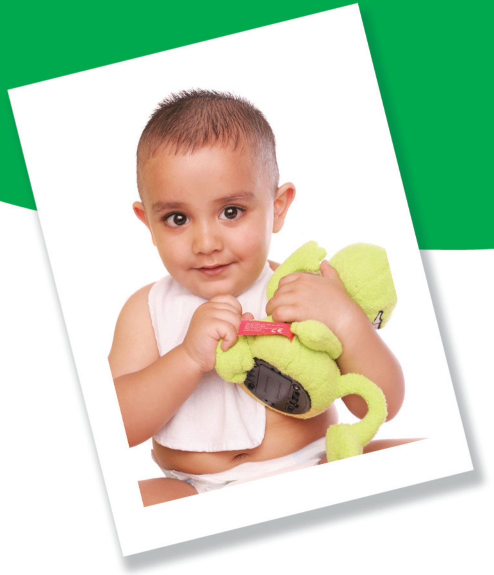

There are many different charitable acts (Sadaqah) in the eyes of Allah (SWT). Your involvement in the Born In Bradford. Inshallah will be seen as an act of continuous charity. Prophet Muhammad (PBUH) reminded us of the importance of ongoing reward for certain acts. In the following

> *"When a person dies, his deeds come to an end. But there are three things done by him for which he continues to receive the reward from Allah.... he leaves behind some knowledge which is continuously benefiting the people." Muslim*

Also, by getting involved in the Born in Bradford project you will help in not only improving the health of your own family but others as well.

You can help in a number of ways –

- By actually agreeing to take part in the study if asked.
- By supporting our fundraising campaign in any way you can.
- By sending us your views and suggestions

Please contact us if you have any questions or would like to know more about the project.

Authors:
Moulana Mohammed Arshad,
Maryam Riaz - Muslim Chaplains

BORNINBRADFORD
Project Office, Bradford Royal Infirmary,
Duckworth Lane, Bradford, BD9 6RJ
tel: 01274 364474 email: bib@bradfordhospitals.nhs.uk

Registered Charity No: 1061753

Patrons: Imran Khan, Chancellor, University of Bradford
Baroness Lockwood of Dewsbury

www.borninbradford.nhs.uk

bib
BORNINBRADFORD
For a Healthy Future

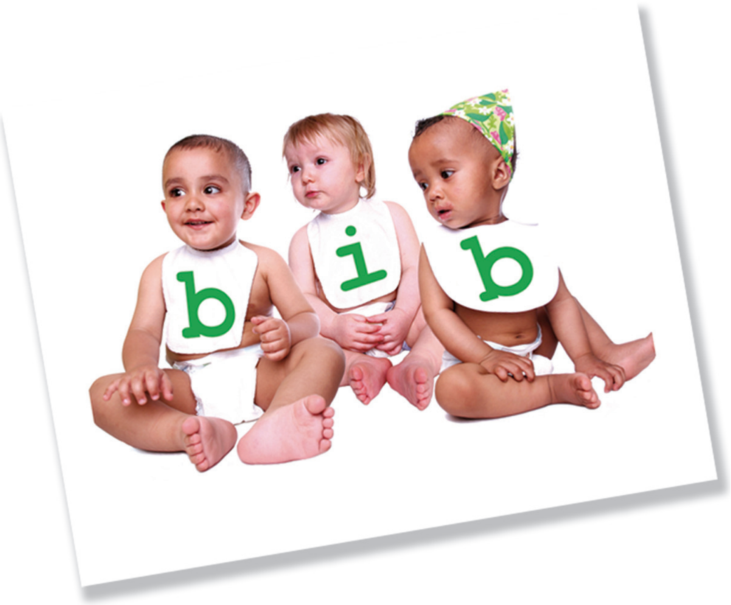

The Islamic Perspective

Bradford Teaching Hospitals NHS Foundation Trust

www.borninbradford.nhs.uk

Leaflet Inner.1 21/9/06 15:51 Page 2

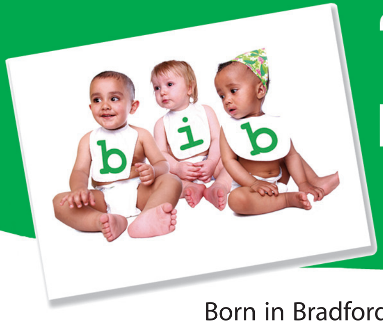

"Born in Bradford will form one of the world's biggest studies into why our children fall ill."

Born in Bradford is a project designed to try and find out the causes of diseases and how they can be prevented.

Medical research has always played an important role in the lives of Muslims. This dates back to the middle ages when Muslims attained the highest scientific achievements in medicine and healthcare. The reason behind this success was Islam itself. The Holy Qur'an and Hadith (teachings of the Prophet Muhammad (PBUH) have encouraged the gaining of knowledge as Prophet Muhammad (PBUH) has stated,

> *"It is necessary for every Muslim to gain knowledge"*
> *Ibn Majah and Behaqui*

According to the following Hadith, Muslim scholars agree the Sunnah (practice) of Prophet Muhammad (PBUH) is to seek treatment.

> *"Allah has not made a disease without appointing a remedy for it, with the exception of one disease, namely old age."*
> *Bukhari*

Allah (SWT) has directed that we seek treatments for our illness. This means seeking help and advice from doctors and other trained professionals. The Born In Bradford project will look into seeking the best treatment for the health of our children.

> Bradford has a growing population and is currently the fifth largest metropolitan district in the UK. However, we have high levels of illness and disease.
>
> In the short time it takes you to read these sentences another Bradford child is being treated for ill-health. Sometimes the solution is easily found. Sometimes it is not. Sadly, more babies die in Bradford than the national average.

Islam promotes the rights and responsibilities of an individual upon themselves and toward others. One of the responsibilities is to your children in fulfilling their right to good health and education. As prophet Mohammad said:

> *"Every one of you is a Shepherd (responsible) and every one of you will be answerable for his flock."*
> *Bukhari*

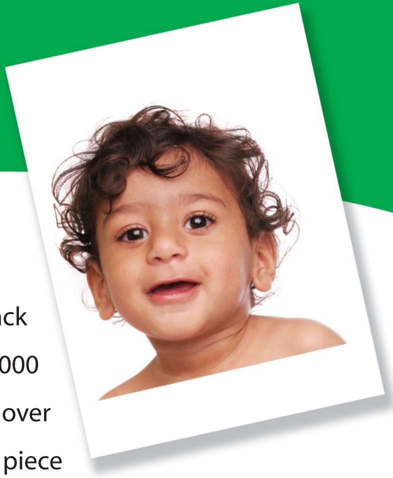

Born in Bradford will track the lives of around 10,000 babies born in the city over the next three years and piece together a picture of each child. from pregnancy, through childhood, until they become adults. We will look at factors such as their genes, diet, lifestyle, schooling, neighbourhood and upbringing.

By pinpointing the causes of disease, we will have the key to improving not only the health of Bradford people, but the health of others around the country and around the world.

b i b
BORNINBRADFORD
For a Healthy Future
