# Supplementary material for: Born in Bradford, a cohort study of babies born in Bradford, and their parents: Protocol for the recruitment phase
Source: BMC Public Health. 2008 Sep 23;8:327. doi: 10.1186/1471-2458-8-327 (PMC2562385; doi:10.1186/1471-2458-8-327)
Supplement: Additional file 3 — Polish, Slovakian and Czech Leaflet. Summary leaflet handed out to Polish, Slovakian and Czech mothers explaining the study and offering the help of an interpreter if they wish to take part. [file 1471-2458-8-327-S3.doc]

**Welcome to Bradford Royal Infirmary Maternity Unit.**

I expect you have noticed some women waiting for their Glucose Tolerance Test have been approached by staff other than nurses from the hospital. These members of staff are asking them to take part in a new study called Born in Bradford.

This project aims to track the progress of 10,000 babies from birth, through childhood into adulthood. Mothers-to-be who attend this clinic are being asked to take part. The information we collect will help us to discover the causes of childhood diseases and how we can work together to improve the health of all babies born in Bradford

In order to agree to make a decision about this it is important we can explain in full what the project is about and what happens when you decide to take part. Unfortunately we are sorry we do not have an interpreter here at the present time that can help us to explain what the study is about and answer any questions you may have.

However, if you are interested in taking part we can arrange a special appointment here in the Maternity Unit for you to meet with someone from the study and an interpreter to talk through what happens if you sign up for the project. It will also give you a chance to ask any questions you may have about the project.

If you are interested please contact the project office 01274 364474


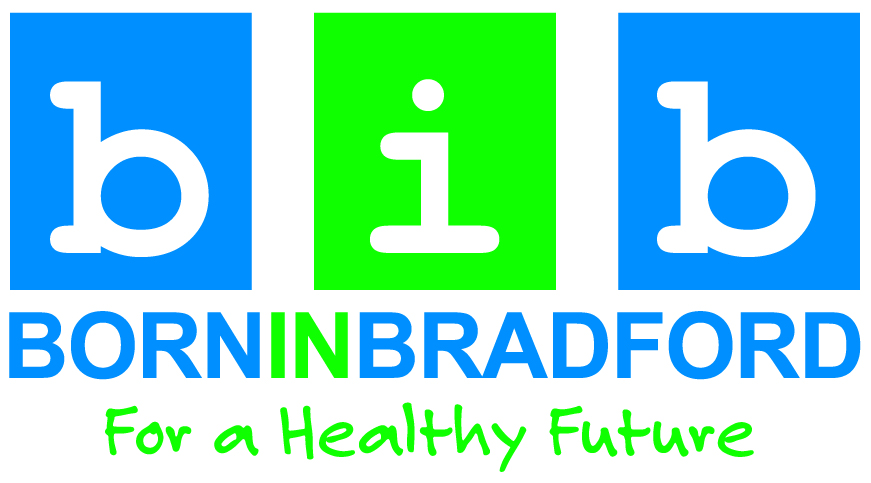
**POLISH**

Witamy na oddziale położniczym szpitala Bradford Royal Infirmary.

Być może zauważyły Panie, że pracownicy szpitala, inni niż pielęgniarki, zwracają się do niektόrych pacjentek oczekujących na wykonanie testu glukozowego, w sprawie wzięcia udziału w nowych badaniach o nazwie ‘Urodzony w Bradford’.

Celem projektu jest obserwacja rozwoju 10 000 dzieci od momentu urodzenia, przez dzieciństwo, aż po dorosłość. Przyszłe matki uczęszczające do naszej przychodni proszone są o wzięcie udziału w badaniach. Zebrane informacje pomogą nam odkryć przyczyny chorόb wieku dziecięcego, oraz wypracować metody wspόlnej pracy na rzecz poprawy zdrowia wszystkich dzieci urodzonych w Bradford.

Aby mogły Panie podjąć decyzję dotyczącą udziału w projekcie, ważne jest abyśmy mogli w pełni wyjaśnić istotę projektu oraz co za sobą pociąga wzięcie udziału w badaniach. Przykro nam, ale niestety w tym momencie nie mamy tłumacza, ktόry mόgłby pomόc nam wyjaśnić na czym polegają badania, oraz odpowiedzieć na wszelkie pytania.

Jednak, jeżeli są Panie zainteresowane wzięciem udziału w projekcie, możemy zorganizować specjalne spotkanie tutaj na oddziale położniczym, podczas ktόrego będą Panie mogły poznać osoby prowadzące badania, oraz przy pomocy tłumacza porozmawiać na temat uczestnictwa. Spotkanie może także stać się okazją do wzięcia udziału w projekcie, jeżeli będą sobie Panie tego życzyć.

Jeżeli są Panie zainteresowane, prosimy o kontakt

**SLOVAKIAN**


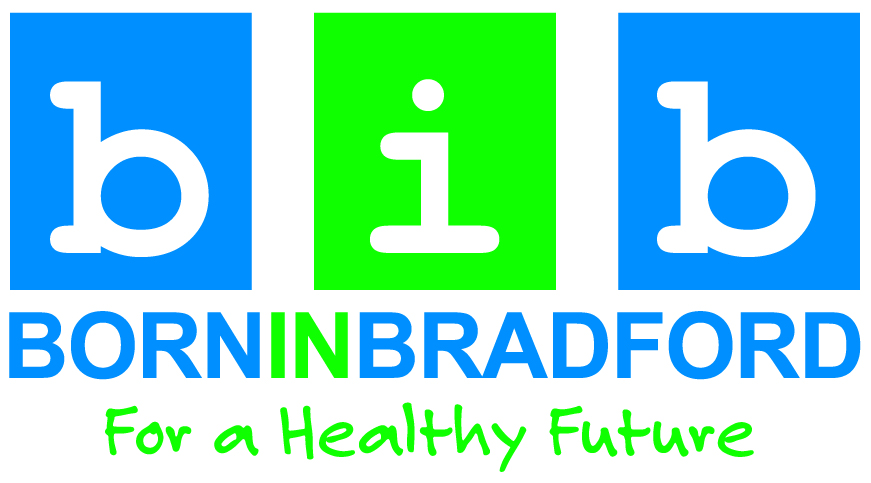
**Vitajte v Bradford Royal Infirmary Maternity Unit (Bradfordskej kráľovskej pôrodnici).**

Predpokladám, že ste si všimli, že s niektorými ženami, ktoré čakajú na Glukóznu skúšku tolerancie (Glucose Tolerance Test), hovoril iný personál než zdravotné sestry z nemocnice. Tento personál ich prosí, aby sa zúčastnili nového výskumu, ktrorý sa volá Born in Bradford (Narodený v Bradforde).

Cielom tohoto projektu je zaznamenať vývoj 10,000 bábätiek od narodenia, cez detstvo až do dospelosti. Budúce matky, ktoré chodia na túto kliniku boli požiadané, aby sa na tom podielali. Získané informácie nám pomôžu objaviť príčiny detských chorôb a ako môžeme spolupracovať na zlepšení zdravia všetkých bábätiek narodených v Bradforde.

K tomu, aby sa dal súhlas k zúčastneniu sa tohoto projektu je dôležité, aby sme plne vysvetlili o čom tento projekt je a čo sa stane keď sa na ňom rozhodnete podielať. Je nám bohužaľ ľúto, že tu momentálne nemáme tlmočníka, ktorý by nám mohol pomôcť vysvetliť o čom je tento výskum a odpovedať na akékoľvek otázky, ktoré máte.

Keď ale máte záujem na tom podielať sa, môžeme zabezpečit schôdzku tu na Maternity Unit, aby ste sa stretli s niekym s výskumu a s tlmočníkom a porozprávali sa o tom čo to všetko obnáša, keby ste sa zúčastnili a ak si prajete tiež mať možnosť zúčastniť sa.

Keď máte záujem kontaktujte nás

**
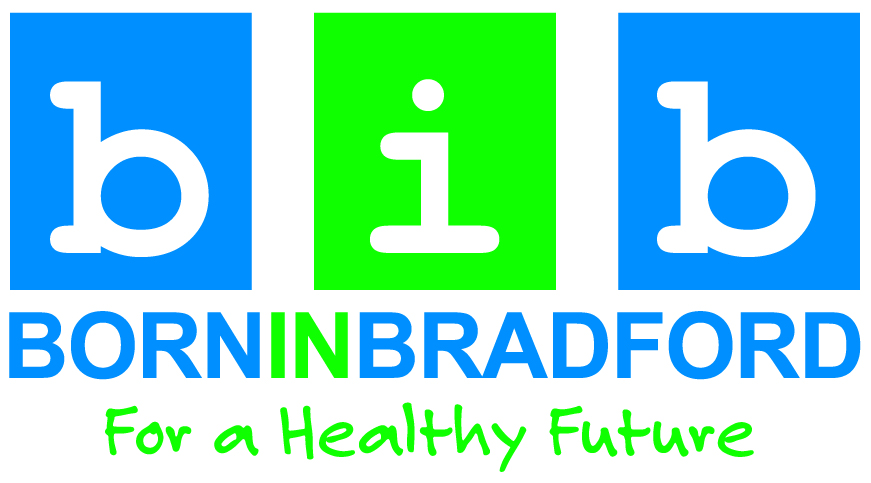
**

**CZECH**

Vítejte v Bradford Royal Infirmary Maternity Unit (Bradfordský královský porodnici).

Předpokládám, že jste si všimli, že s některými ženami, které čekají na Glukózní skoušku tolerance (Glucose Tolerance Test), mluvil jiný personal než zdravotní sestry z nemocnice. Tenhle personal je prosí, aby se zoučastnili novýho výskumu, ktrerý s jmenuje Born in Bradford (Narozen v Bradfordu).

Cílem tohodle projektu je zaznamenat vývoj 10,000 dětí od narození, přes dětství až do dospělosti. Budoucí matky, které chodí na tuhle kliniku byli požádané, aby se na tom podíleli. Získané informace nám pomůžou objevit příčiny dětských nemocí a jak můžeme spolupracovat na zlepšení zdraví všech dětí narozených v Bradfordu.

K tomu, aby se dal souhlas k zoučastnění se tohodle projektu je důležité, aby jsme plně vysvětlili o čem tenhle projekt je a co se stane když se na něj rozhodnete podílet. Je nám bohužel líto, že tady momentálně nemáme tlumočníka, který by nám mohl pomoct vysvětlit o čem je tenhle výskum a odpovědět na jakékoliv otázky, které máte.

Když ale máte zájem na tom podílet se, můžeme zabezpečit schůzku tady na Maternity Unit, aby jste se setkala s někým s výskumu a s tlumočníkem a promluvili si co to všechno obnáší, kdyby jste se zoučastnili a když si přejete mít možnost zoučastnit se.

Když máte zájem kontaktujte nás
