# Supplementary material for: Born in Bradford, a cohort study of babies born in Bradford, and their parents: Protocol for the recruitment phase
Source: BMC Public Health. 2008 Sep 23;8:327. doi: 10.1186/1471-2458-8-327 (PMC2562385; doi:10.1186/1471-2458-8-327)
Supplement: Additional file 4 — Mothers' Consent Form. Mothers' Consent Form. [file 1471-2458-8-327-S4.doc]

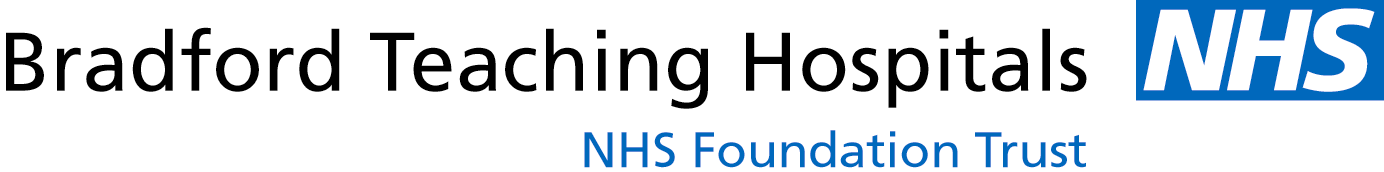

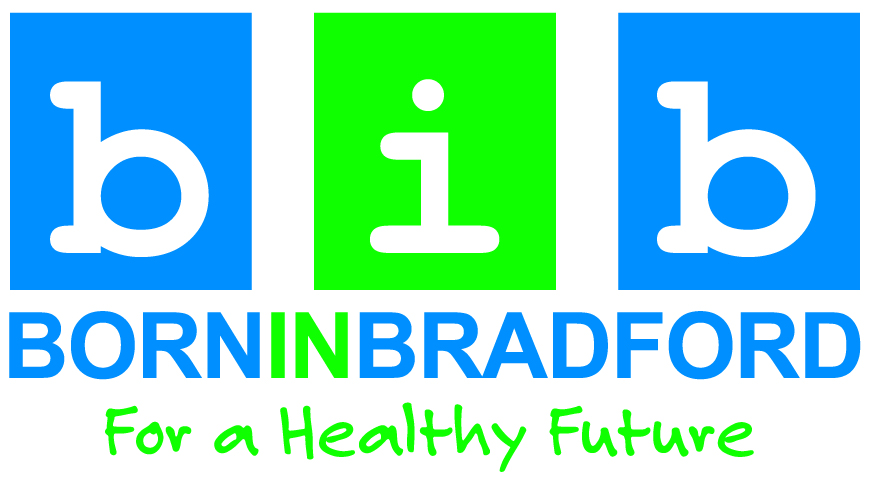


**MOTHER’S CONSENT FORM**

# A copy of this consent form will be retained in your hospital case notes

I confirm I have read and understood the information sheet dated 26^th^ June 2007 (Version 7) and have had the opportunity to ask questions. I also understand that my participation and that of my child is voluntary and that we are free to withdraw at any time, without giving any reason, without our medical care or legal rights being affected

I understand that researchers working for the Born in Bradford project may be look at sections of my medical records and the educational and medical records of my child. Researchers working on ethically approved linked studies both inside and outside Europe, after approval by the Executive Group and ethics committee, may also have access to information. Some of these countries do not have the same data protection laws as in the UK, however I understand the information cannot be linked to me.

I understand that my child’s details may be passed onto the ethically approved Office of National Statistics and that this will require the permission of the local Data Advisory Group.

I agree that one of the project researchers can approach me in the future.

I agree to give biological samples, which may include blood, saliva and urine for use in the above study which may be stored for future use. If I wish to withdraw from the study in the future I agree these samples may be retained and used unless I specifically request they are destroyed, in which case we will make every effort to do so and ensure that no further analysis is conducted on your samples".

I agree to blood being taken from my baby’s umbilical cord after delivery. I understand that my baby’s biological samples and mine will be stored. I understand the Born in Bradford team and their research partners in the UK and both inside and outside Europe will use these samples and that I will not be given the results.

………………………… ……………. …………………..

Print name of participant date signature

………………………… ……………. …………………..

Print name of person taking consent date signature

(if different from Study Administrator)

…

……………………… ……………. …………………..

Print name of Study Administrator date signature
