# Supplementary material for: Born in Bradford, a cohort study of babies born in Bradford, and their parents: Protocol for the recruitment phase
Source: BMC Public Health. 2008 Sep 23;8:327. doi: 10.1186/1471-2458-8-327 (PMC2562385; doi:10.1186/1471-2458-8-327)
Supplement: Additional file 5 — Mother's Questionnaire. Baseline questionnaire completed by mothers who agree to take part in Born in Bradford. [file 1471-2458-8-327-S5.pdf]

Draft

Study ID [ ][ ][ ][ ][ ] [P][ ]

Born in Bradford - Mothers' Questionnaire

To be completed by interviewer:

Interviewer's Number [ ][ ] - [ ][ ] *(2 initials - 2 numbers e.g. AN 01)*

1. Date Completing this questionnaire?

[ ][ ] / [ ][ ] / [2][0][ ][ ]

d d / m m / y y y y

***2. What language(s) was used for administering the questionnaire?**

☐ English ☐ Mirpuri/Punjabi ☐ Urdu

Any other language (please write in)

***3. Was an Interpreter used?**

☐ No ☐ Hospital/Study Interpreter ☐ Family Member/Friend

(To be measured by interviewer)

4. Height (Cms) [ ][ ][ ] . [ ]

5. Weight [ ][ ][ ] . [ ][ ]
(Kilos) (Grms)

***6. Triceps** (Cms) [ ][ ] . [ ] ☐ Not able to take

***7. Arm circumference** (Cms) [ ][ ] . [ ] ☐ Not able to take

8. How old were you when you had your first period?

[ ][ ] [ ][ ] ☐ Don't Know

y y m m

9. Will this be your first child? ☐ Yes ☐ No

9a) If no:- what month and year were each of your previous children born in ?
- starting with the eldest:

	Month	Year
First child		
Second child		
Third child		
Fourth child		
Fifth child		
(add birth dates of all other children)		

Draft

Section A - Where you live

These questions relate to where you are living at present.

***A1. How long have you lived at your current address?** [ ][ ] y y [ ][ ] m m

***A2. In which of these ways does your household occupy this address?**
(Cross ONE box ONLY)

If answers yes to any of the three * questions, please go to A2a). If not go to A3

- ☐ Buying it with the help of a mortgage or loan
- ☐ Owns outright
- ☐ *Rents it
- ☐ *Lives here rent free (including rent free in relatives/friends property excluding squatting)
- ☐ *Pays part rent and part mortgage (shared ownership)
- ☐ Don’t know
- ☐ Squatting

***A2a) If A2 was answered - Rents it: Lives rent free or pays part rent and part mortgage - ask who is your landlord?**
(Cross ONE box ONLY)

- ☐ Private Landlord or Letting Agency, Another individual
- ☐ Housing Association, Housing Co-operative, Charitable Trust
- ☐ Local Authority/Council
- ☐ Relative or friend (before you lived here) of a household member
- ☐ Employer (individual) of a household member
- ☐ Employer (organisation) of a household member
- ☐ Another Organisation
- ☐ Don't Know

A3) How many bedrooms does your household have, including bedsitting rooms and spare bedrooms?

Enter number of bedrooms [ ][ ]

Section B - Who you live with?

B1. What ages are those, including yourself, who live in your household or accommodation? [If age not known, please give best estimate]

Is there anybody:-

Age	Number of males	Number of females
Under 2 years		
between 2 -15 years		
between 16 - 17 years		
between 18 - 64 years		
65 years and over		

B2. Are you: (Cross ONE box ONLY)

- ☐ Married (first marriage)
- ☐ Re-married
- ☐ Single (never married)
- ☐ Separated (but still legally married)
- ☐ Divorced
- ☐ Widowed

B3. Are you: (Cross ONE box ONLY)

- ☐ Living with baby's father
- ☐ Living with another partner
- ☐ Not living with a partner – but in a relationship (eg. partner living abroad or in another propery)
- ☐ Not living with a partner and not in a relationship

Section C - About you, your family and your baby's father and his family

C1. What country were you and your baby's father born in?

(Cross ONE box ONLY in each column).

Country	You	Country	Baby's father
England	☐	England	☐
Northern Ireland	☐	Northern Ireland	☐
Scotland	☐	Scotland	☐
Wales	☐	Wales	☐
Channel Islands	☐	Channel Islands	☐
Isle of Man	☐	Isle of Man	☐
Republic of Ireland	☐	Republic of Ireland	☐
Czech Republic	☐	Czech Republic	☐
Poland	☐	Poland	☐
Slovakia	☐	Slovakia	☐
Bangladesh	☐	Bangladesh	☐
India	☐	India	☐
Pakistan	☐	Pakistan	☐
Sri Lanka	☐	Sri Lanka	☐
Philippines	☐	Philippines	☐
Don’t know	☐	Don’t know	☐

You - Other (Please write in)

Baby's father - Other (Please write in)

***C2. To be asked if not born in the UK**
How old were you when you moved to the UK?

Age in Years [ ][ ] [ ][ ]
y y m m

If answered Pakistan for you and/or baby's father in C1 go to C3.
If not Pakistan for either person then go to C4.

C3) Were you and/or baby's father born in Mirpur District?
(Cross ONE box ONLY in each row)

**You**	☐ Yes	☐ No	☐ Don't Know
**Baby's father**	☐ Yes	☐ No	☐ Don't Know

***C3a) If yes, which town or village?**

You (Please write in) ☐ Don't Know

Baby's father (Please write in) ☐ Don't Know

***C3b) Do you know the name of your's and baby's father's Biraderi?**
(Interviewer - consult list of Biraderi if necessary).

You (Please write in) ☐ Don't Know

Baby's father (Please write in) ☐ Don't Know

Draft

*C4. What country were your mother and father born in?
(Cross ONE box ONLY in each column).

Country	Your Mother	Country	Your Father
England	☐	England	☐
Northern Ireland	☐	Northern Ireland	☐
Scotland	☐	Scotland	☐
Wales	☐	Wales	☐
Channel Islands	☐	Channel Islands	☐
Isle of Man	☐	Isle of Man	☐
Republic of Ireland	☐	Republic of Ireland	☐
Czech Republic	☐	Czech Republic	☐
Poland	☐	Poland	☐
Slovakia	☐	Slovakia	☐
Bangladesh	☐	Bangladesh	☐
India	☐	India	☐
Pakistan	☐	Pakistan	☐
Sri Lanka	☐	Sri Lanka	☐
Philippines	☐	Philippines	☐
Don’t know	☐	Don’t know	☐

Your mother - Other (Please write in)

☐☐☐☐☐☐☐☐☐☐☐☐☐☐☐☐☐☐☐☐☐☐☐☐☐☐☐☐☐

Your father - Other (Please write in)

☐☐☐☐☐☐☐☐☐☐☐☐☐☐☐☐☐☐☐☐☐☐☐☐☐☐☐☐☐

If answered Pakistan for your mother or father in C4 go to C5 if not Pakistan then go to C6

C5) Were your mother and father born in Mirpur district?
(Cross ONE box ONLY)

**Your mother**	☐ Yes	☐ No	☐ Don't Know
**Your father**	☐ Yes	☐ No	☐ Don't Know

1 2 3 4 5

Draft

C5a) If yes, which town or village?

Your mother - (Please write in) ☐ Don't Know

Your father - (Please write in) ☐ Don't Know

C5b) Do you know the name of your mother's and father's Biraderi?

Your mother - (Please write in) ☐ Don't Know

Your father - (Please write in) ☐ Don't Know

C6 What country were your grandparents born in?

(Cross ONE box ONLY in each column).

Country	Your mother's mother	Your mother's father	Your father's mother	Your father's father
England	☐	☐	☐	☐
Northern Ireland	☐	☐	☐	☐
Scotland	☐	☐	☐	☐
Wales	☐	☐	☐	☐
Channel Islands	☐	☐	☐	☐
Isle of Man	☐	☐	☐	☐
Republic of Ireland	☐	☐	☐	☐
Czech Republic	☐	☐	☐	☐
Poland	☐	☐	☐	☐
Slovakia	☐	☐	☐	☐
Bangladesh	☐	☐	☐	☐
India	☐	☐	☐	☐
Pakistan	☐	☐	☐	☐
Sri Lanka	☐	☐	☐	☐
Philippines	☐	☐	☐	☐
Don’t know	☐	☐	☐	☐

Your mother's mother - Other (Please write in)

Your mother's father - Other (Please write

Your father's mother - Other (Please write in)

Your father's father - Other (Please write in)

If answered Pakistan in C6 for where any of your grandparents were born ask C7:
If not go to C8.

Answer this if your grandparents were born in Pakistan If not go to C8.

C7. Were your grandparents born in Mirpur district? (Cross ONE box ONLY in each row)

**Your mothers mother**	☐ Yes	☐ No	☐ Don't Know
**Your mother's father**	☐ Yes	☐ No	☐ Don't Know
**Your father's mother**	☐ Yes	☐ No	☐ Don't Know
**Your father's father**	☐ Yes	☐ No	☐ Don't Know

C7a) If yes, which town or village ?

Your mother's mother - (Please write in) ☐ Don't Know

Your mother's father - (Please write in) ☐ Don't Know

Your father's mother - (Please write in) ☐ Don't Know

Your father's father - (Please write in) ☐ Don't Know

C7b) Do you know the name of your grandparent's Biraderies?

Your mothers mother - (Please write in) ☐ Don't Know

Your mothers father - (Please write in) ☐ Don't Know

Your fathers mother - (Please write in) ☐ Don't Know

Your fathers father - (Please write in) ☐ Don't Know

C8. What country were the parents of your baby's father born in?

(Cross ONE box ONLY in each column).

Country	Mother of baby's father	Country	Father of baby's father
England	☐	England	☐
Northern Ireland	☐	Northern Ireland	☐
Scotland	☐	Scotland	☐
Wales	☐	Wales	☐
Channel Islands	☐	Channel Islands	☐
Isle of Man	☐	Isle of Man	☐
Republic of Ireland	☐	Republic of Ireland	☐
Czech Republic	☐	Czech Republic	☐
Poland	☐	Poland	☐
Slovakia	☐	Slovakia	☐
Bangladesh	☐	Bangladesh	☐
India	☐	India	☐
Pakistan	☐	Pakistan	☐
Sri Lanka	☐	Sri Lanka	☐
Philippines	☐	Philippines	☐
Don’t know	☐	Don’t know	☐

Mother of baby's father - Other (Please write in)

Father of baby's father - Other (Please write in)

If answered Pakistan in C8 for where the mother of the baby's father or father of the baby's father was born ask C9: If not then go to C10.

Answer this if the mother of the baby's father or father of the baby's father was born in Pakistan

C9. Was the mother of the baby's father / father of the baby's father born in Mirpur ? (Cross ONE box ONLY in each row)

Mother of baby's father ☐ Yes ☐ No ☐ Don't Know

Father of baby's father ☐ Yes ☐ No ☐ Don't Know

C9a) If yes, which town or village ?

Mother of baby's father - (Please write in) ☐ Don't Know

Father of baby's father - (Please write in) ☐ Don't Know

C9b) Do you know the name of your baby's father's parents' Biraderies?

Mother of baby's father - (Please write in) ☐ Don't Know

Father of baby's father - (Please write in) ☐ Don't Know

Returning to you;

C10. To which of these groups do you consider you belong? (Cross ONE box ONLY)

☐ White
☐ Asian or Asian British
☐ Mixed ethnic group
☐ Chinese
☐ Black or Black British
☐ Other

C10a) If Answered C10 as White what do you consider your cultural background? (Cross ONE box ONLY)

☐ British ☐ Irish

Any other white background (please write in)

Draft

C10b) If Answered C10 as Mixed ethnic group what do you consider your cultural background? (Cross ONE box ONLY)

- ☐ White and Black Caribbean
- ☐ White and Black African
- ☐ White and Indian
- ☐ White and Pakistani
- ☐ White and Bangladeshi
- ☐ White and Indian Caribbean
- ☐ White and African-Indian

Any other mixed background (please write in)

C10c) If answered C10 as Black or Black British what do you consider your cultural background? (Cross ONE box ONLY)

- ☐ Caribbean
- ☐ African

Any other Black background (please write in)

C10d) If answered C10 as Asian or Asian British what do you consider your cultural background? (Cross ONE box ONLY)

- ☐ Indian
- ☐ Pakistani
- ☐ Bangladeshi
- ☐ Indian Caribbean
- ☐ African-Indian

Any other Asian background (please write in)

C10e) If answered C10 as Chinese what do you consider your cultural background? (Cross ONE box ONLY)

- ☐ Chinese
- ☐ Japanese
- ☐ Filipino
- ☐ Vietnamese

Any Chinese or other background (please write in)

C10f) If answered C10 as Other what do you consider your cultural background? (please write in)

Draft

1 2 3 4 5

Section D - Your Family

These questions are about you and your family and about baby's father and his family.

D1. Are you related to the father of your baby other than by marriage? For example are you cousins? (Cross ONE box ONLY)

☐ Yes ☐ No ☐ Don't Know

D1a) If yes, how are you related to the father of your baby? e.g. 1st cousin, 2nd cousin (Cross ONE box ONLY)

☐ 1st Cousin
☐ 1st Cousin, once removed
☐ Second Cousin
☐ Other related by blood
☐ Other related by marriage
☐ Don't know

D2. Were your parents related? For example were they cousins? (Cross ONE box ONLY)

☐ Yes ☐ No ☐ Don't Know

D2a) If yes, how were your parents related? (Cross ONE box ONLY)

☐ 1st Cousins
☐ 1st Cousins, once removed
☐ Second Cousins
☐ Other related by blood
☐ Other related by marriage
☐ Don't know

D3. Were the parents of the father of your baby related? For example were they cousins? (Cross ONE box ONLY)

☐ Yes ☐ No ☐ Don't Know

D3a) If yes, how were they related? (Cross ONE box ONLY)

☐ 1st Cousins
☐ 1st Cousins, once removed
☐ Second Cousins
☐ Other related by blood
☐ Other related by marriage
☐ Don't know

Interviewer: If answered yes in D1, please complete a family tree (on a separate form after you have completed this section. Do not change questions D1 to D3 after the family tree is completed).

D4. Was a family tree completed? ☐ Yes ☐ No

Section E Education

E1. What is the highest educational qualification you have? (Cross ONE box ONLY)

- ☐ 1 + 0 levels/CSEs/GCEs (any grades)
- ☐ 5 + 0 levels, 5+ CSEs (grade 1) 5 + GCSEs (grades A-C), School Certificate
- ☐ 1 + A levels/AS levels
- ☐ 2 + A levels, 4 + AS levels, Higher School Certificate
- ☐ NVQ Level 1, Foundation GNVQ
- ☐ NVQ Level 2,Intermediate GNVQ
- ☐ NVQ Level 3, Advanced GNVQ
- ☐ NVQ Levels 4-5, HNC, HND
- ☐ First Degree (e.g. BA, BSc)
- ☐ Higher Degree (e.g. MA, PhD, PGCE Post-graduate certificates/diplomas)
- ☐ Other qualifications (e.g. City and Guilds, RSA/OCR, BTEC/Edexcel)
- ☐ Overseas qualification ***(If obtained in Pakistan go to E1a, If obtained in another country go to E1b)***
- ☐ No Qualifications
- ☐ Don't know

E1a) If your highest educational qualification was obtained in Pakistan please indicate: (Cross ONE box ONLY)

- ☐ Second School Certificate (SSC) Matriculation (Metric)
- ☐ Diploma in Commerce
- ☐ Higher Secondary (HSC) Cert/Intermediate Humanities, Pre-Eng or Pre-Medical/Science Streams
- ☐ Certificate from Board of Technical Education
- ☐ Diploma from Board of Technical Education
- ☐ Final Apprenticeship Certificate/Grade 2 Skilled
- ☐ Vocational Institute Diploma/Grade 3 Skilled Worker Certificate
- ☐ Batchelor Degree (4 year) in generally professional fields (excluding Bachelor of Education)
- ☐ Batchelor of Arts/Commerce/Engineering/Science/Technology (Pass and Honours)
- ☐ Postgraduate Eg Masters degree/PhD
- ☐ Don't know

E1b) If your highest educational qualification was not obtained in the UK or in Pakistan, please specify what that qualification was and which country it was obtained in.

Qualification

Country

E2. What is baby's father's highest educational qualification? (Cross ONE box ONLY)

- ☐ 1 + 0 levels/CSEs/GCEs (any grades)
- ☐ 5 + 0 levels, 5+ CSEs (grade 1) 5 + GCSEs (grades A-C), School Certificate
- ☐ 1 + A levels/AS levels
- ☐ 2 + A levels, 4 + AS levels, Higher School Certificate
- ☐ NVQ Level 1, Foundation GNVQ
- ☐ NVQ Level 2,Intermediate GNVQ
- ☐ NVQ Level 3, Advanced GNVQ
- ☐ NVQ Levels 4-5, HNC, HND
- ☐ First Degree (e.g. BA, BSc)
- ☐ Higher Degree (e.g. MA, PhD, PGCE Post-graduate certificates/diplomas)
- ☐ Other qualifications (e.g. City and Guilds, RSA/OCR, BTEC/Edexcel)
- ☐ Overseas qualification ***(If obtained in Pakistan go to E2a, If obtained in another country go to E2b)***
- ☐ No Qualifications
- ☐ Don’t know

E2a) If his highest educational qualification was obtained in Pakistan please indicate (Cross ONE box ONLY)

- ☐ Second School Certificate (SSC) Matriculation (Metric)
- ☐ Diploma in Commerce
- ☐ Higher Secondary (HSC) Cert/Intermediate Humanities, Pre-Eng or Pre-Medical/Science Streams
- ☐ Certificate from Board of Technical Education
- ☐ Diploma from Board of Technical Education
- ☐ Final Apprenticeship Certificate/Grade 2 Skilled
- ☐ Vocational Institute Diploma/Grade 3 Skilled Worker Certificate
- ☐ Batchelor Degree (4 year) in generally professional fields (excluding Bachelor of Education)
- ☐ Batchelor of Arts/Commerce/Engineering/Science/Technology (Pass and Honours)
- ☐ Postgraduate Eg Masters degree/PhD
- ☐ Don’t know

E2b) If his highest educational qualification was not obtained in the UK or Pakistan, please specify what the qualification was and which country it was obtained in.

Qualification

Country

Draft

Section F Your Current Employment

F1. Are you currently a full time student? ☐ Yes ☐ No

F2. Are you currently working? ☐ Yes ☐ No (If Yes, go to F3)

F2a) If No - Have you ever worked? ☐ Yes ☐ No (If No, go to F12)

F2b) If yes to F2a - how long ago did you stop working?

Years ☐☐ Months ☐☐

If stopped less than one month ago record weeks - (round up to nearest week) Weeks ☐☐

F3. Are you currently on Maternity/Sick Leave? ☐ Yes ☐ No

F4. Were/are you self employed? ☐ Yes ☐ No

F4a) If no to F4 - If an employee, what type of industry/company do/did you work for?

F5. What was/is your job title?

F5b) How many people work at the place that you usually work?

☐ 1-2 ☐ 3-24 ☐ 25-499 ☐ 500+ (Cross ONE box ONLY)

F5c) Are/were you a: (Cross ONE box ONLY)

☐ Manager ☐ Supervisor ☐ Other Employee

F6. Please list the 2 or 3 main tasks you perform/performed at work?

F7. How many hours do/did you work in a typical week?

Indicate number of hours ☐☐.☐

1 2 3 4 5

Draft

Only answer F8-F11 if currently working or stopped working less than one year ago.

F8. How long have/had you done this job?

Years ☐☐ Months ☐☐

F9. Where is/was your main place of work? (Cross ONE box ONLY)

☐ Work mainly at or from home ☐ No regular place of work

If neither of the above ask 10 and 10a. Everyone should be asked F11.

F10. What is/was your main place of work?

Street

Town

Postcode

F10a. How do/did you usually travel to work?

Cross ONE box ONLY indicating what is/was the longest part, by distance, of your usual journey to work.

- ☐ Work mainly at or from home
- ☐ Train
- ☐ Bus, minibus or coach
- ☐ Motor cycle, scooter or moped
- ☐ Driving a car or van
- ☐ Passenger in a car or van
- ☐ Taxi
- ☐ Bicycle
- ☐ On foot
- ☐ Other

Other - (please write in)

F11. How many days in a typical week do/did you go to work?

(Enter 0 if works mainly at or from home)

Days ☐☐

About baby's father

*F12. Which best describes the sort of work the baby's father does?

If not in work now, please **cross ONE box ONLY** to show what work he did in his last main job.

- ☐ Modern professional occupations
- ☐ Clerical and intermediate occupations
- ☐ Senior managers or administrators
- ☐ Technical and craft occupations
- ☐ Semi-routine manual and service occupations
- ☐ Routine manual and service occupations
- ☐ Middle or junior managers
- ☐ Traditional professional occupations
- ☐ Self Employed
- ☐ Student/in training
- ☐ Does not work – long term unemployed/ill health (one year or over).
- ☐ Don’t know

***F13. Can you tell me which of these credits/allowances/benefits you and your husband/partner receive?**

(Please cross ALL that apply)

- ☐ Child Benefit
- ☐ Child Tax Credit
- ☐ Working Tax Credit (Formerly Working Family Tax Credit and Disabled Person's Tax Credit)
- ☐ Income Support
- ☐ Disability Living Allowance (including Disabled Person's Tax Credit)
- ☐ Income tested Job Seekers Allowance (Unemployment)
- ☐ Housing Benefit/Rent Rebate/Council Tax Benefit
- ☐ Incapacity Benefit (Replaces Invalidity and NI Sickness Benefit)
- ☐ Pension Credit
- ☐ Carer's allowance (was Invalid Care Allowance)
- ☐ None
- ☐ Don't know
- ☐ Does not wish to answer

Any Other State Benefit Please specify below

***F14. This table shows income in weekly, monthly and annual amounts.**

Which of the amounts on this list represents you and your husband/partner's, total income from all jobs, (full and part time), all tax credits, all benefits and all other sources and earnings after tax when all income is added together. (Cross ONE box ONLY)

Weekly Income after Tax	Monthly Income after tax	Annual Income after Tax	
Less than £25	Less than £108	less than £1,299	☐
£25 - £39	£109 - £175	£1,300 - £2,099	☐
£40 - £59	£176 - £259	£2,100 - £3,099	☐
£60 - £79	£260 - £350	£3,100 - £4,199	☐
£80 - £99	£351 - £433	£4,200 - £5,199	☐
£100 - £124	£434 - £542	£5,200 - £6,499	☐
£125 - £149	£543 - £650	£6,500 - £7,799	☐
£150 - £179	£651 - £775	£7,800 - £9,299	☐
£180 - £209	£776 - £917	£9,300 - £10,999	☐
£210 - £259	£918 - £1,125	£11,000 - £13,499	☐
£260 - £299	£1,126 - £1,333	£13,500 - £15,999	☐
£300 - £379	£1,334 - £1,667	£16,000 - £19,999	☐
£380 - £479	£1,668 - £2,083	£20,000 - £24,999	☐
£480 - £577	£2,084 - £2,500	£25,000 – £29,999	☐
£578 - £769	£2,501 - £3,333	£30,000 - £39,999	☐
£770 - £962	£3,334 - £4,167	£40,000 - £49,999	☐
£963 - £1,154	£4,168 - £5,000	£50,000 - £59,999	☐
£1,155 - £1,346	£5,001 - £5,833	£60,000 - £69,999	☐
£1,347 - £1,538	£5,834 - £6,667	£70,000 - £79,999	☐
£1,539 or more	£6,668 or more	£80,000 or more	☐
Does not wish to answer ☐		Don't know	☐

The next few questions are about the sorts of things that some people have but which many people have difficulty finding the money for.

***F15. Do you or you and your husband/partner have?**
(Cross ONE box ONLY in each row)

	Yes	I/we would like this but can't afford it at this moment	I/we do not want/need this at the moment	Does not wish to answer	Don't know
**a)** A holiday from home for at least one week once a year (not including staying with relatives in their home)	☐	☐	☐	☐	☐
**b)** Friends or family who call for a drink or meal at your house at least once a month	☐	☐	☐	☐	☐
**c)** Two pairs of all weather shoes	☐	☐	☐	☐	☐
**d)** Enough money to keep your home in a decent state of decoration	☐	☐	☐	☐	☐
**e)** Household contents Insurance	☐	☐	☐	☐	☐
**f)** Money to make regular savings of £10 a month or more for rainy days or retirement	☐	☐	☐	☐	☐
**g)** Money to replace any worn out furniture	☐	☐	☐	☐	☐
**h)** Money to replace or repair major electrical goods such as a refrigerator or a washing machine when broken	☐	☐	☐	☐	☐
**i)** A small amount of money to spend each week on yourself (not on your family)	☐	☐	☐	☐	☐
**j)** A hobby or leisure activity	☐	☐	☐	☐	☐
**k)** In winter are you able to keep your home warm enough	☐	☐	☐	☐	☐

***F16. Sometimes people are not able to pay every bill when it falls due. May I ask, are you up to date with the bills on this list or are you behind with any of them?**

Interviewer: Show card with list of bills

F16a) Are you up to date with all these bills? (Cross ONE box ONLY)

☐ Yes ☐ No ☐ Don't Know ☐ Does not wish to answer

F16b) If no, which ones are you behind with? (Cross ALL that apply)

- ☐ Electricity Bill
- ☐ Gas
- ☐ Other fuel bills like coal or oil
- ☐ Council tax
- ☐ Insurance Policies
- ☐ Telephone Bill
- ☐ Television/video/DVD rental or hire purchase
- ☐ Other hire purchase payments
- ☐ Water rates

***F17. These questions apply if you have any children living in your household now.** (Cross ONE box ONLY in each row)

	Yes	Would like to have this but cannot afford this at the moment	Children do not want/need this at the moment	Does not apply
**a)** Are there enough bedrooms for every child of 10 or over of a different sex to have their own bedroom.	☐	☐	☐	☐

The following questions apply to your children living with you

	Yes	Would like to have this but cannot afford this at the moment	Children do not want/need this at the moment	Does not apply
**b)** Does your child/children have leisure equipment or a bicycle	☐	☐	☐	☐
**c)** Does your child/children have celebrations on special occasions such as birthdays, or religious festivals	☐	☐	☐	☐
**d)** Does your child/do your children go swimming at least once a month	☐	☐	☐	☐
**e)** Does your child/children do A hobby or leisure activity	☐	☐	☐	☐
**f)** Does your child/children have friends round for tea or a snack once a fortnight	☐	☐	☐	☐

F18. If you have any children age under 6 who are not in School
(Cross ONE box ONLY)

	Yes	Would like to have this but cannot afford this at the moment	Children do not want/need this at the moment	Does not apply
Does your child/children go to a toddler group/nursery/playgroup at least once a week	☐	☐	☐	☐

F19. If your child/children is/are over age 6 or in school.
(Cross ONE box ONLY)

	Yes	Would like to have this but cannot afford this at the moment	Children do not want/need this at the moment	Does not apply
Does your child/children go on school trips	☐	☐	☐	☐

F20. For children of all ages (Cross ONE box ONLY)

	Yes	No	Does not apply
Does your child/children have an outdoor space or facilities nearby where they can play safely	☐	☐	☐

F21. How well would you say you or you and your husband/partner are managing financially these days. Would you say you are? (Cross ONE box ONLY)

☐ Living comfortably
☐ Doing alright
☐ Just about getting by
☐ Finding it quite difficult
☐ Finding it very difficult
☐ Does not wish to answer

F22. Compared to a year ago, how would you say you and your husband/partner are doing financially now? (Cross ONE box ONLY)

☐ Better off
☐ Worse off
☐ About the same
☐ Does not wish to answer

Section G - Smoking/Alcohol/Drug Use

We apologise if any questions in this section cause offence - we are asking everyone the same questions but we realise some religions do not permit certain things.

SMOKING

G1. Have you ever regularly smoked cigarettes; that is at least one cigarette a day? (Cross ONE box ONLY)

☐ Yes for more than 1 year ☐ Yes for less than 1 year ☐ No

If NO, go to question G4

G1a) How old were you when you started smoking cigarettes?

Age: ☐☐ Years old ☐ Don't Remember

G2. Do you smoke cigarettes nowadays? ☐ Yes ☐ No (Cross ONE box ONLY)

G2a) If no, when did you stop smoking?

Age: ☐☐ Years old ☐ Don't Remember

G3. How many cigarettes do/did you smoke during pregnancy, or in the three months before pregnancy? (Cross ONE box ONLY in each row)

	None	1-5 a day	6-10 a day	11-20 a day	Over 20 a day
**a)** 3 months before	☐	☐	☐	☐	☐
**b)** First 3 months of pregnancy	☐	☐	☐	☐	☐
**c)** Since the beginning of 4th month	☐	☐	☐	☐	☐

G4. During pregnancy have you been exposed to other peoples' cigarette smoke at work or at home and if Yes, for how many hours per day approx? (Cross ONE box ONLY)

☐ Yes ☐ No ☐ Less than 1 hour per day/occasionally

If yes - Hours ☐☐

G5. Have you used any other tobacco products like Paan during pregnancy, or in the 3 months before pregnancy? Interviewer: please show list of possible products.
(Cross ONE box ONLY)

☐ Yes ☐ No ☐ Don't Know

If No, Don't Know or you don't remember go to question G6

***G5a) If yes please identify which ones and how many you smoke/chew etc., (relevant to point in pregnancy)**

3 Months before pregnancy

	Daily	Weekly	Monthly	Rarely	If 1+ per week, how many per week
	☐	☐	☐	☐	
	☐	☐	☐	☐	
	☐	☐	☐	☐	
	☐	☐	☐	☐	

First 3 months of pregnancy

	Daily	Weekly	Monthly	Rarely	If 1+ per week, how many per week
	☐	☐	☐	☐	
	☐	☐	☐	☐	
	☐	☐	☐	☐	
	☐	☐	☐	☐	

From beginning of 4th month to now

	Daily	Weekly	Monthly	Rarely	If 1+ per week, how many per week
	☐	☐	☐	☐	
	☐	☐	☐	☐	
	☐	☐	☐	☐	
	☐	☐	☐	☐	

G6. Have you used any drugs like marijuana or ecstasy during pregnancy or in the three months before pregnancy? (Cross ONE box ONLY)

☐ Yes ☐ No ☐ Don't Know

If No, Don't Know or you don't remember go to question G7

Draft

***G6a) If yes please identify which ones and how often you have taken them (relevant to point in pregnancy)**

3 Months before pregnancy	Daily	Weekly	Monthly	Rarely	If 1+ per week, how many per week
	☐	☐	☐	☐	
	☐	☐	☐	☐	
	☐	☐	☐	☐	
	☐	☐	☐	☐	
**First 3 months of pregnancy**					
	☐	☐	☐	☐	
	☐	☐	☐	☐	
	☐	☐	☐	☐	
	☐	☐	☐	☐	
**From beginning of 4th month to now**					
	☐	☐	☐	☐	
	☐	☐	☐	☐	
	☐	☐	☐	☐	
	☐	☐	☐	☐	

ALCOHOL

G7. Did you drink any alcohol during your pregnancy or in the 3 months before? (Cross ONE box ONLY)

☐ Yes ☐ No ☐ Don't Remember If NO or don't remember go to Section H

G7a) Did you drink any alcohol in the 3 months before pregnancy?
(Cross ONE box ONLY)

☐ Yes, Once per week or more ☐ Yes, occasionally ☐ No ☐ Don't remember

If NO or don't remember go to question G7d)

G7b) If once per week or more, what is the weekly average and maximum number of units in a week?

	Average number of units per week	Maximum units at one time
Beer/Lager	☐☐	☐☐
Wine	☐☐	☐☐
Spirits	☐☐	☐☐
Other	☐☐	☐☐
Don't remember	☐	☐

G7c) In the 3 months before pregnancy how often did you consume 5 or more units of alcohol on one occasion? (Cross ONE box ONLY)

☐ Everyday ☐ 1-3 times a month

☐ Nearly every day ☐ Rarely

☐ 1-4 times/week ☐ Never

G7d) Did you drink any alcohol in the first 3 months of pregnancy?
(Cross ONE box ONLY)

☐ Yes, Once per week or more ☐ Yes, occasionally ☐ No ☐ Don't remember

If NO or don't remember go to section G7g)

G7e) If once per week or more, what is the average and maximum number of units in a week?

	Average number of units per week	Maximum units at one time
Beer/Lager	☐☐	☐☐
Wine	☐☐	☐☐
Spirits	☐☐	☐☐
Other	☐☐	☐☐
Don't remember	☐	☐

G7f) In the first 3 months of pregnancy how often did you consume 5 or more units of alcohol on one occasion? (Cross ONE box ONLY)

- ☐ Every day or more often
- ☐ Nearly every day
- ☐ 1-4 times/week
- ☐ 1-3 times a month
- ☐ Rarely
- ☐ Never

G7g) Did you drink any alcohol from the beginning of the 4th month until now of your pregnancy? (Cross ONE box ONLY)

☐ Yes, Once per week or more ☐ Yes, occasionally ☐ No ☐ Don’t remember

If NO or don't remember go to section H

G7h) If once per week or more, what is the average and maximum number of units in a week?

	Average number of units per week	Maximum units at one time
Beer/Lager	☐☐	☐☐
Wine	☐☐	☐☐
Spirits	☐☐	☐☐
Other	☐☐	☐☐
Don't remember	☐	☐

G7i) Since the beginning of the 4th month of your pregnancy how often did you consume 5 or more units of alcohol on one occasion?
(Cross ONE box ONLY)

- ☐ Every day or more often
- ☐ Nearly every day
- ☐ 1-4 times/week
- ☐ 1-3 times a month
- ☐ Rarely
- ☐ Never

BREADS AND BREAD PRODUCTS

H1. During the last 4 weeks, on average how many slices/pieces of the following did you eat per week?

	Total Slices/Pieces	No. eaten as toast
**a)** White bread incl baguette	☐☐	☐☐
**b)** Brown bread incl granary, multiseed, best of both, 50/50.	☐☐	☐☐
**c)** Baps/rolls/teacake	☐☐	☐☐
**d)** Crumpets	☐☐	☐☐
**e)** Pizza (1 slice)	☐☐	
**f)** Roti/Chappatis	☐☐	
**g)** Naan, pitta bread, bagel	☐☐	☐☐
**h)** Paratha	☐☐	
Other bread products e.g. wraps, croissants, pancakes, flatbreads, English muffin	☐☐	☐☐

Other please write in

Draft

CAFFEINATED DRINKS

H2. During the last 4 weeks of pregnancy, on average, how many cups or mugs of the following drinks would you have per day or per week?

(Glass is 200 ml Cup is 200 ml 1 Mug = 2 cups.
If less than 1 per day enter weekly average)

How many cups of: ?	Per day	Per Week
**a)** Instant coffee (Caffeinated)	☐☐	☐☐
**b)** Instant coffee (De-caffeinated)	☐☐	☐☐
**c)** Filter/cafetiere coffee (Caffeinated)	☐☐	☐☐
**d)** Filter/cafetiere coffee (De-caffeinated)	☐☐	☐☐
**e)** Tea (Caffeinated)	☐☐	☐☐
**f)** Tea (De-caffeinated)	☐☐	☐☐
**g)** Kashmiri tea (Caffeinated)	☐☐	☐☐
**h)** Kashmiri tea (De-caffeinated)	☐☐	☐☐
**i)** Herbal/fruit teas (Caffeinated)	☐☐	☐☐
**j)** Herbal/fruit teas (De-caffeinated)	☐☐	☐☐
**k)** Cola (regular, with sugar Caffeinated)	☐☐	☐☐
**l)** Cola (regular, with sugar De-caffeinated)	☐☐	☐☐
**m)** Diet or sugar free cola (Caffeinated)	☐☐	☐☐
**n)** Diet or sugar-free cola (De-caffeinated)	☐☐	☐☐

1 2 3 4 5

Draft

Draft

SUPPLEMENTS/VITAMINS

H3. Have you taken any dietary supplements including vitamins or iron tablets in the last 4 weeks of pregnancy? (Cross ONE box ONLY)

☐ Yes ☐ No ☐ Don't Remember

H3a) If Yes, which:

	Daily	5-6 per week	2-4 per week	Once a week	Less Often
Vitamin C	☐	☐	☐	☐	☐
Vitamin D	☐	☐	☐	☐	☐
Vitamin E	☐	☐	☐	☐	☐
Iron	☐	☐	☐	☐	☐

Other vitamins and dietary supplements, please write in below:

Don't Know ☐

If multivitamins:

Pregnacare	☐	☐	☐	☐	☐
Sanatogen prenatal	☐	☐	☐	☐	☐

Other multivitamins, please write in below:

Don't Know ☐

Draft

1 2 3 4 5

Section I Water Consumption

***I1. On a typical day how much of the following do you drink?**

	At home		At work/study		Elsewhere	
a) Tap water	Glasses per day:	☐☐	Glasses per day:	☐☐	Glasses per day:	☐☐
b) Bottled water (Includes water cooler)	Glasses per day:	☐☐	Glasses per day:	☐☐	Glasses per day:	☐☐
c) Tea (any sort)	Cups per day:	☐☐	Cups per day:	☐☐	Cups per day:	☐☐
d) Coffee	Cups per day:	☐☐	Cups per day:	☐☐	Cups per day:	☐☐
e) Squash (Including any other drinks made with tap water)	Glasses per day:	☐☐	Glasses per day:	☐☐	Glasses per day:	☐☐

I2. Do you filter the water you drink at home? (Cross ONE box ONLY)

☐ Yes ☐ No ☐ Don't Know

I3. Do you filter the water you drink at work? (Cross ONE box ONLY)

☐ Yes ☐ No ☐ Don't Know ☐ N/A

I4. In a typical week while you have been pregnant how often and for how long do you undertake the following?
(if you do not do any then fill in 0)

	Times per week	Minutes each time
Shower	☐☐	☐☐☐
Bath	☐☐	☐☐☐
Swim	☐☐	☐☐☐

Section J General Health

Interviewer to give questionnaire for this section to be self-completed.

We should like to know if you have had any medical complaints and how your health has been in general, over the past few weeks. Please answer ALL the questions on the following pages simply by putting a cross by the answer which you think most nearly applies to you. Remember that we want to know about present and recent complaints, not those that you had in the past. It is important that you try to answer ALL the questions.

Cross ONE box ONLY for each question - have you:

J1a. Been feeling perfectly well and in good health?

☐ Better than usual ☐ Same as usual ☐ Worse than usual ☐ Much worse than usual

J1b. Been feeling in need of a good tonic?

☐ Not at all ☐ No more than usual ☐ Rather more than usual ☐ Much more than usual

J1c. Been feeling run down and out of sorts?

☐ Not at all ☐ No more than usual ☐ Rather more than usual ☐ Much more than usual

J1d. Felt that you are ill?

☐ Not at all ☐ No more than usual ☐ Rather more than usual ☐ Much more than usual

J1e. Been getting any pains in your head?

☐ Not at all ☐ No more than usual ☐ Rather more than usual ☐ Much more than usual

J1f. Been getting a feeling of tightness or pressure in your head?

☐ Not at all ☐ No more than usual ☐ Rather more than usual ☐ Much more than usual

J1g. Been having hot or cold spells?

☐ Not at all ☐ No more than usual ☐ Rather more than usual ☐ Much more than usual

J2a. Lost much sleep over worry?

☐ Not at all ☐ No more than usual ☐ Rather more than usual ☐ Much more than usual

J2b. Had difficulty in staying asleep once you are off?

☐ Not at all ☐ No more than usual ☐ Rather more than usual ☐ Much more than usual

J2c. Felt constantly under strain?

☐ Not at all ☐ No more than usual ☐ Rather more than usual ☐ Much more than usual

J2d. Been getting edgy and bad- tempered?

☐ Not at all ☐ No more than usual ☐ Rather more than usual ☐ Much more than usual

J2e. Been getting scared or panicky for no good reason?

☐ Not at all ☐ No more than usual ☐ Rather more than usual ☐ Much more than usual

J2f. Found everything getting on top of you?
☐ Not at all ☐ No more than usual ☐ Rather more than usual ☐ Much more than usual

J2g. Been feeling nervous and strung-up all the time?
☐ Not at all ☐ No more than usual ☐ Rather more than usual ☐ Much more than usual

J3a. Been managing to keep yourself busy and occupied?
☐ More so than usual ☐ Same as usual ☐ Rather less than usual ☐ Much less than usual

J3b. Been taking longer over the things you do?
☐ Quicker than usual ☐ Same as usual ☐ Longer than usual ☐ Much longer than usual

J3c. Felt on the whole you were doing things well?
☐ Better than usual ☐ About the same as usual ☐ Less well than usual ☐ Much less well

J3d. Been satisfied with the way you've carried out your tasks?
☐ More satisfied ☐ About the same as usual ☐ Less satisfied than usual ☐ Much less satisfied

J3e. Felt that you are playing a useful part in things?
☐ More so than usual ☐ Same as usual ☐ Less useful than usual ☐ Much less than usual

J3f. Felt capable of making decisions about things?
☐ More so than usual ☐ Same as usual ☐ Rather less so than usual ☐ Much less capable

J3g. Been able to enjoy your normal day-to-day activities?
☐ More so than usual ☐ Same as usual ☐ Less so than usual ☐ Much less than usual

J4a. Been thinking of yourself as a worthless person?
☐ Not at all ☐ No more than usual ☐ Rather more than usual ☐ Much more than usual

J4b. Felt that life is entirely hopeless?
☐ Not at all ☐ No more than usual ☐ Rather more than usual ☐ Much more than usual

J4c. Felt that life isn't worth living?
☐ Not at all ☐ No more than usual ☐ Rather more than usual ☐ Much more than usual

J4d. Thought of the possibility that you might make away with yourself?
☐ Definitely not ☐ I don’t think so ☐ Has crossed my mind ☐ Definitely have

J4e. Found at times you couldn't do anything because your nerves were too bad?
☐ Not at all ☐ No more than usual ☐ Rather more than usual ☐ Much more than usual

J4f. Found yourself wishing you were dead and away from it all?
☐ Not at all ☐ No more than usual ☐ Rather more than usual ☐ Much more than usual

J4g. Found that the idea of taking your own life kept coming into your mind?
☐ Definitely not ☐ I don’t think so ☐ Has crossed my mind ☐ Definitely has

Section K Exercise

Interviewer to give questionnaire for this section to be self-completed

K1. Please tell us about the type and amount of physical activity involved in your paid work. (Cross ONE box ONLY)

☐ I am not in paid employment

☐ I spend most of my time at work sitting (such as in an office)

☐ I spend most of my time at work standing or walking. However my work does not require much intense physical effort (e.g. shop assistant; hairdresser; childminder)

☐ My work involves definite physical effort including handling of heavy objects and use of tools (e.g. cleaner; hospital nurse; gardener, postal delivery worker)

☐ My work involves vigorous physical activity including handling of very heavy objects.

K2. During the last week how many hours did you spend on each of the following activities? (Cross only one box in each row)

	**None**	**Some but less than one hour**	**1 hour but less than 3 hours**	**3 hours or more**
**a)** Physical exercise such as swimming, jogging, aerobics, tennis, gym workout etc	☐	☐	☐	☐
**b)** Cycling, including cycling to work and during leisure time	☐	☐	☐	☐
**c)** Walking, including walking to work, shopping, for pleasure etc	☐	☐	☐	☐
**d)** Housework/childcare	☐	☐	☐	☐
**e)** Gardening/DIY (Do it Yourself)	☐	☐	☐	☐

K3. How would you describe your usual walking pace?

☐ Slow pace ☐ Steady average pace

☐ Brisk pace ☐ Fast pace

<u>Please return to the interviewer' - 'Thank you for completing this questionnaire</u>

Draft

Section Interviewer's feedback

L1. Was anyone present with Mother during the interview? (Cross ONE box ONLY)

☐ Yes ☐ No ☐ Part of interview

L1a) If yes or part of interview: who was present? (Cross ALL that apply)

☐ Baby's father ☐ Mother's friend

☐ Mother's mother ☐ Relative

☐ Mother's father ☐ Child

Other (please write in)

L2 Was a transliteration used to administer the questionnaire? ☐ Yes ☐ No ☐ Partially
(Cross ONE box ONLY)

L3 Were there any problems in completing this interview? ☐ Yes ☐ No

L3a) If yes, what were the problems

L4 Do you feel confident with the answers provided? ☐ Yes ☐ No

L4a) If no, why are you not confident?

COMPLETED QUESTIONNAIRE - CHECKED BY STUDY ADMINISTRATOR ☐ Yes

ALSO CHECKED: ☐ M Diet ☐ J General Health ☐ K Exercise

BY: Name

Interviewer Number
(if applicable)

		-		

1 2 3 4 5

Draft

Section M Your diet

Questionnaire about your diet

This short questionnaire asks you about the food you have eaten over the last four weeks of your pregnancy. You may not eat all the foods given or you may find that some of the foods you eat are not included – please do not worry but complete all of the question asked.

Please do not leave any of the lines blank and answer every question even if you are uncertain.

INSTRUCTIONS ABOUT HOW TO COMPLETE THE QUESTIONS

Please put a cross in each box to show how often you have eaten each food item. E.g. if you eat 4 slices of white bread a day – cross the box as shown below

FOOD ITEM	HOW OFTEN HAVE YOU EATEN THIS IN THE LAST 4 WEEKS?						
	Rarely or never	Less than 1 a Week	Once a Week	2-3 times a Week	4-6 times a Week	1-2 times a Day	3-4 times a Day	5+ a Day
White bread	0	1	2	3	4	5	X 6	7

If you make a mistake and cross the wrong box, just cross out and enter the cross in the correct box.
E.g. If you cross you had fruit juice 3 times a day when you meant 3 times a week just cross out the '3-4 times a day' answer and cross the '2-3 times a week' box.

FOOD ITEM	HOW OFTEN HAVE YOU EATEN THIS IN THE LAST 4 WEEKS?						
	Rarely or never	Less than 1 a Week	Once a Week	2-3 times a Week	4-6 times a Week	1-2 times a Day	3-4 times a Day	5+ a Day
Fruit juice (not cordial or squash)	0	1	2	X 3	4	5	~~X~~	7

M1. The following questions ask about some food and drinks you might have consumed during the last 4 weeks of your pregnancy. Do not be concerned if some things you eat or drink are not mentioned.

Please cross how often you eat at least ONE portion of the following foods & drinks: (a portion includes: a packet of crisps, a serving of chips, one bowl of cereal). **(Please cross ONE box ONLY, but answer EVERY line even if you don't eat that food)**

	Rarely or never	Less than 1 a Week	Once a Week	2-3 times a Week	4-6 times a Week	1-2 times a Day	3-4 times a Day	5+ Times a Day
**a)** Chips	☐	☐	☐	☐	☐	☐	☐	☐
**b)** Roast or fried potatoes, hash browns or potato waffles	☐	☐	☐	☐	☐	☐	☐	☐
**c)** Fibre or bran-rich wheat breakfast cereal, like Weetabix, Fruit 'n Fibre, Bran flakes	☐	☐	☐	☐	☐	☐	☐	☐
**d)** Oat cereals including muesli, porridge, crunchy oats, instant hot oats	☐	☐	☐	☐	☐	☐	☐	☐
**e)** Other breakfast cereals like cornflakes, rice krispies, Cheerios	☐	☐	☐	☐	☐	☐	☐	☐
**f)** Crispbread, like Ryvita	☐	☐	☐	☐	☐	☐	☐	☐
**g)** Pasta or noodles (also pot noodles, tinned spaghetti)	☐	☐	☐	☐	☐	☐	☐	☐
**h)** Savouries like Yorkshire puddings, dumplings, pakoras or bhajia	☐	☐	☐	☐	☐	☐	☐	☐
**i)** Potato crisps	☐	☐	☐	☐	☐	☐	☐	☐
**j)** Other salted savoury snacks like tortilla chips, Wotsits, Quavers, Bombay mix	☐	☐	☐	☐	☐	☐	☐	☐
**k)** Cakes, buns, gateaux, doughnuts, muffins	☐	☐	☐	☐	☐	☐	☐	☐
**l)** Sweet pastries like fruit pies, Danish pastries, custard/curd tarts, croissants	☐	☐	☐	☐	☐	☐	☐	☐
**m)** Chocolate bars and chocolate coated biscuits e.g. Twix, Kit-Kat, Dairy milk bar	☐	☐	☐	☐	☐	☐	☐	☐
**n)** Sweet biscuits like digestive, custard creams, gingernut, shortbread	☐	☐	☐	☐	☐	☐	☐	☐

Draft

M2. The following questions ask about types of meat and fish you might have consumed over the last 4 weeks of your pregnancy. Please cross how often you eat at least ONE portion of the following:

	Rarely or never	Less than 1 a Week	2-3 times a Week	4-6 times a Week	7+ times a week
**Whole meats**					
**a)** Beef - steaks, roasts, joints, or chops (not in sauce)	☐	☐	☐	☐	☐
**b)** Pork - steaks, roasts, joints, or chops (not in sauce)	☐	☐	☐	☐	☐
**c)** Lamb, mutton or goat	☐	☐	☐	☐	☐
**d)** Chicken or Turkey - steaks, roasts, joints, portions (not in batter, sauce or breadcrumbs)	☐	☐	☐	☐	☐
**Processed meats/meat**					
**e)** Meat sausages e.g. Walls or chipolata	☐	☐	☐	☐	☐
**f)** Beef burgers, either home cooked or takeaway	☐	☐	☐	☐	☐
**g)** Kebabs	☐	☐	☐	☐	☐
**h)** Hot dog, frankfurter or saveloy	☐	☐	☐	☐	☐
**i)** Bacon rashers	☐	☐	☐	☐	☐
**j)** Meat pies and pastries (sausage roll, pasties, meat samosa, steak/meat pie)	☐	☐	☐	☐	☐
**k)** Chicken/turkey nuggets, Kiev, turkey or chicken burgers, chicken pies, or in batter or breadcrumbs	☐	☐	☐	☐	☐
**l)** Ham	☐	☐	☐	☐	☐
**m)** Cured/dried sausage e.g. Chorizo, Salami	☐	☐	☐	☐	☐
**Meat dishes**					
**n)** Chicken or turkey with sauce e.g. curry, stir-fry, casserole	☐	☐	☐	☐	☐
**o)** Beef, lamb or goat in sauce e.g. curry, stew, Shepherd's pie, Bolognese sauce, Chilli con carne, Lasagne	☐	☐	☐	☐	☐
**p)** Pork in sauce e.g. stew, casserole or stir-fry	☐	☐	☐	☐	☐
**q)** Gravy made with pan or meat juices (not instant)	☐	☐	☐	☐	☐
**Fish**					
**r)** White fish in batter or breadcrumbs, like 'fish 'n chips'	☐	☐	☐	☐	☐
**s)** White fish not in batter or breadcrumbs e.g. cod in parsley sauce, fish curry (marsala fish), fish pie	☐	☐	☐	☐	☐
**t)** Tinned tuna	☐	☐	☐	☐	☐
**u)** Fresh or tinned oily fish like sardines, mackerel, salmon, trout (not tuna)	☐	☐	☐	☐	☐
**v)** Smoked fish, like smoked mackerel, kippers or smoked salmon	☐	☐	☐	☐	☐
**w)** Salted/dried fish e.g. 'Bombay duck'/bummalo	☐	☐	☐	☐	☐

M3. If eaten in the last 4 weeks of pregnancy how did you mainly cook the following?
Please enter only one cross on each line for cooking method. Cross yes if mainly eaten very well done, crispy or heavily browned as shown.

	Did not eat	Don't know or take-away	Grill	Fry	Roast	BBQ	Well done? Yes	No
**a)** Beef - steaks, roasts, joints, or chops (not in sauce)	☐	☐	☐	☐	☐	☐	☐	☐
**b)** Beef burgers, either home cooked or takeaway	☐	☐	☐	☐	☐	☐	☐	☐
**c)** Pork - steaks, roasts, joints, or chops (not in sauce)	☐	☐	☐	☐	☐	☐	☐	☐
**d)** Lamb, mutton or goat - steaks, roasts, joints, or chops (not in sauce)	☐	☐	☐	☐	☐	☐	☐	☐
**e)** Chicken or Turkey-steaks, roasts, joints, portions (not in batter, sauce or breadcrumbs)	☐	☐	☐	☐	☐	☐	☐	☐
**f)** Meat Sausages e.g. Walls or chipolata	☐	☐	☐	☐	☐	☐	☐	☐
**g)** Bacon rashers, chops or bacon ribs	☐	☐	☐	☐	☐	☐	☐	☐
**h)** White fish fillets or steaks e.g. cod or haddock NOT in batter	☐	☐	☐	☐	☐	☐	☐	☐
**i)** Oily fish fillets or steaks e.g. salmon, mackerel, trout	☐	☐	☐	☐	☐	☐	☐	☐

M4. a. Are you familiar with the "5 a day" recommendations for fruit and vegetables?

☐ Yes ☐ No

b. Do you consume 5 portions of fruit and vegetables per day?
(Please place a CROSS in ONE box ONLY)

☐ Always ☐ Sometimes ☐ Never

(Please place a CROSS in ONE box ONLY)
M5. Where does most of your advice about healthy eating during pregnancy come from?

☐ Family members ☐ GPs/Doctors

☐ Friends ☐ Midwife/Health Visitor

☐ Magazines/Newspapers ☐ Other

☐ Books

Thank you for completing this questionnaire - please leave it in the place indicated.
