# Supplementary material for: Born in Bradford, a cohort study of babies born in Bradford, and their parents: Protocol for the recruitment phase
Source: BMC Public Health. 2008 Sep 23;8:327. doi: 10.1186/1471-2458-8-327 (PMC2562385; doi:10.1186/1471-2458-8-327)
Supplement: Additional file 7 — Father's Questionnaire. Self-completion questionnaire given out to fathers who wish to take part in Born in Bradford. [file 1471-2458-8-327-S7.pdf]

51527

Born in Bradford
Husband/Partner Questionnaire 1

Mother's BIB Study ID [ ][ ][ ][ ][ ] [P][ ]

Date Completing this questionnaire? [ ][ ] / [ ][ ] / [2][0][ ][ ]
d d m m y y y y

What language was used for administering the questionnaire?

☐ English ☐ Urdu (Mark answer with cross)

Husband/Partner's Height? [ ][ ][ ].[ ] (Cms)

Fathers Weight? [ ][ ][ ].[ ] (Kilos)

Thank you for agreeing to complete this questionnaire.

1. What age were you when you finished your education? Please CROSS ONE box only

☐ 16 or under ☐ 17 - 19 ☐ 20 or over

2. What job do you do?

Please write in below your job title. (If you do not currently work write in your last main job)

[ ]

3. If you were not born in the UK how old were you when you moved permanently to the UK? [ ][ ] (Years) [ ][ ] (Months)

4. Do you smoke cigarettes nowadays? (Please CROSS ONE box only)

☐ Yes ☐ No

4a) If you answered YES, about how many cigarettes a day do you smoke?

1-5 Cigarettes ☐ 6-10 Cigarettes ☐ 11-20 Cigarettes ☐ Over 20 ☐ Don't Know ☐

5. Do you drink alcohol, for example beer, wine or spirits? Please CROSS ONE box only

☐ Yes ☐ No

5a) If YES, what is, on average, the weekly average number of units you drink?

ONE unit = 1/2 Pint Beer/Lager/Cider, a Small Glass of Wine or a Single measure of spirits.

Add up all the number of units, of any sort of alcohol, in a typical week. [ ][ ] Average number of units per week

Thank you very much for completing this questionnaire. Please return it to the person who asked you to complete it.
