# Supplementary material for: Born in Bradford, a cohort study of babies born in Bradford, and their parents: Protocol for the recruitment phase
Source: BMC Public Health. 2008 Sep 23;8:327. doi: 10.1186/1471-2458-8-327 (PMC2562385; doi:10.1186/1471-2458-8-327)
Supplement: Additional file 8 — Translation and transliteration process for Urdu and Mirpuri versions of questionnaire. Flowchart outlining process involved in developing the Urdu and Mirpuri transliterated versions of the baseline questionnaire. [file 1471-2458-8-327-S8.doc]

**Translation and transliteration process for Urdu and Mirpuri versions of questionnaire**
