# Supplementary material for: Born in Bradford, a cohort study of babies born in Bradford, and their parents: Protocol for the recruitment phase
Source: BMC Public Health. 2008 Sep 23;8:327. doi: 10.1186/1471-2458-8-327 (PMC2562385; doi:10.1186/1471-2458-8-327)
Supplement: Additional file 9 — Routinely collected data. List of routinely collected maternity data to be extracted from eCLipse the Maternity IT system. [file 1471-2458-8-327-S9.doc]

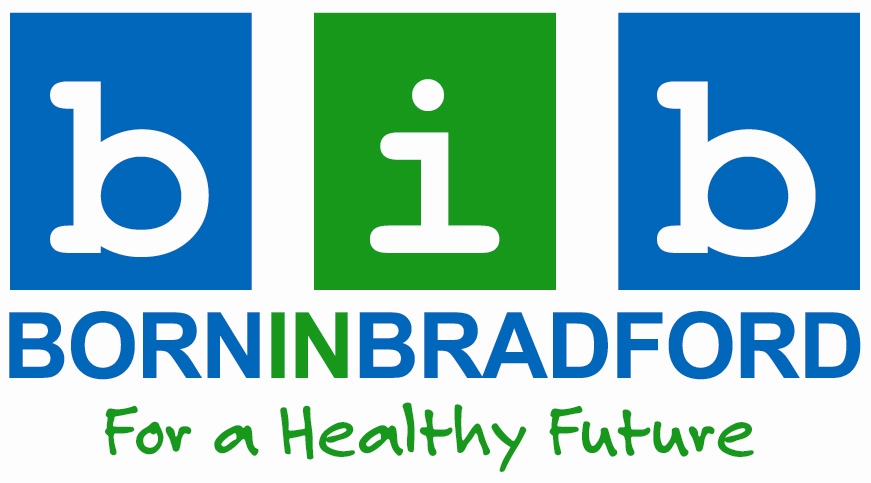

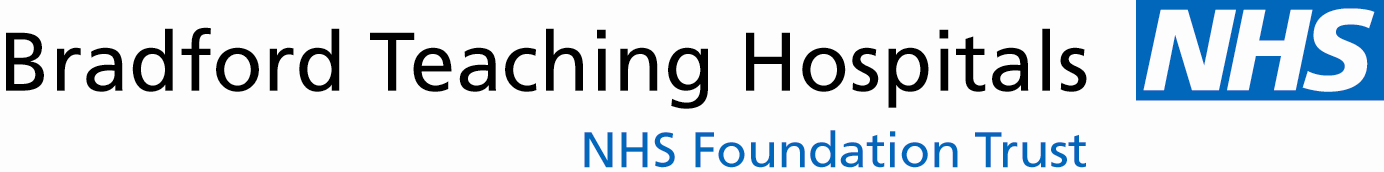


**Routinely collected data**

1. **Booking Details collected by the Midwife at 10 to 14 wks (or later if not referred by this time)**

**Demographic Details**

- - NHS number
  - Name preferred name/family name at birth
  - Address including postcode
  - NOK emergency contact
  - Family Dr name and address
  - Midwife health visitor/interpreter
  - Date of Birth/Age

**Booking Weight and BP BMI**

**BP at 28 & 36**

**Medical & Obstetric History**

- Diabetes/Gestational Diabetes – current pregnancy
- Hypertension
- Preeclampsia – current pregnancy
- Asthma
- Epilepsy
- Mental health problems
- Drugs before 16 wks
- Folic acid use

**Social Details**

- Marital Status single/separated/divorced/married/widowed
- One parent family
- Occupation before home duties
- If working does this mean paid employment?
- If yes need to know more
- Housing – owner / rented / parents / other
- Partner’s occupation
- Partner’s name / address / telephone number / is partner the baby’s father / age of the father

**Ethnicity and Language**

- Languages spoken
- Languages read
- Preferred language/interpreter required
- Ethnic Group – woman and partner
- Place of birth – woman and partner country of origin

**Medical Details**

- Allergies (including latex)

**Miscellaneous**

- Date of booking who booked by
- Number of years UK Domicile – woman
- If less than one year – how many months
- Religion

**Medical & Obstetric History**

- Past illnesses or operations – prior to and including this pregnancy
- Have you had/got:-
- Asthma
- Back problems
- Diabetes
- Epilepsy
- Female circumcision
- Genital Infections
- Gynae history
- Heart Problems
- High blood pressure
- Incontinence
- Kidney/urinary problems
- Liver disease/hepatitis
- Migraine
- Mental health problems
- Psychiatric referral
- TB exposure
- Thyroid problems
- Other
- Operations
- Problems with anaesthetics
- Blood transfusions
- Medication/folic acid
- Vaginal bleeding
- Last cervical smear & result
- Drugs taken this pregnancy – separate information is collected by the one stop team for known drug users
- Do you use drugs - details/Are you receiving treatment for drug addiction/ habits/injections - details
- X-ray other than chest this pregnancy

**Family history**

- TB, Hypertension, diabetes, deafness, blindness, twins, other
- Information from mothers family and the baby’s fathers family
- Only blood relatives, (children, parents, grandparents, siblings, aunts/uncles, cousins
- Thrombosis, eclampsia, mental illness, a disease that runs in the family
- Need for generic counselling
- Stillbirths/miscarriages, SID
- Learning difficulties
- Hearing loss from childhood
- Heart problems from birth
- Abnormalities present at birth
- Consanguinity – none, 1^st^ cousins, 2^nd^ cousins, other
- Is baby’s father a blood relative/ Thalessaemia screening Y/N

**Obstetric History**

- EDD agreed EDD
- Certain LMP
- Previous pregnancies
  - Is current pregnancy with new partner Y/N
  - Date, place of birth, duration of pregnancy, complications, status at birth, gender, birthweight, breast feeding, state of health now, age at and cause of death.
  - Previous gestational Diabetes
  - Previous Pre-eclampsia
- Height
- Weight
- BMI
- Special points for screening
- Anomoly leaflet
- BP

***Other information gathered & activity occurring at booking***

***Lifestyle***

- Diet – halaal/vegetarian/kosher
- Smoking
- Number per day/ if no have you smoked during the past 12 months/ smoking cessation referral
- Alcohol use
- How many units per week / pre pregnancy and currently

**Physical interaction including blood tests**

- Abdominal Examination
- Rubella status
- Urinalysis – first timester
- Bloods explained and accepted by mother, dates of results and actions documented

Leaflet given

FBC, Group Antibody Screen – done at booking and 28 to 30 weeks

FBC repeated at 36 + weeks

If rhesus negative Anti D prophylaxis 28 wks and 34 wks

Syphilis

HIV

Hepatitis B

Electrophoresis

MSU

TT – explained, accepted, date, results, action

Hep C

Time if onset of regular uterine contractions

Spontaneous or induced

Temperature, pulse, Bp

Drugs given in labour

Colour of liquor

Dilatation of cervix

Intensity of contractions

Urinalysis

Time of onset of second stage

Type of delivery

Delivery of placenta and membranes – method/time/completeness

Duration of labour

Duration of ruptured membranes

How membranes were ruptured

Oxytocic drug given

Placental weight

Blood loss

State of perineum

Use of local anaesthetic

Who sutured and material used

Post delivery temperature, pulse and BP

Baby - Midwife

Sex, Weight, PU, Pmec

Temperature, abnormalities

Apgars at 1 minute and 5 minutes

Drugs given

Vitamin K given

Mother

Postnatal urinalysis

Neonatal adverse outcomes (shoulder dysotcia, nerve injury, fracture)

3^rd^ or 4^th^ degree tears/perineal tears
