# Supplementary material for: Born in Bradford, a cohort study of babies born in Bradford, and their parents: Protocol for the recruitment phase
Source: BMC Public Health. 2008 Sep 23;8:327. doi: 10.1186/1471-2458-8-327 (PMC2562385; doi:10.1186/1471-2458-8-327)
Supplement: Additional file 10 — Origin of questions. List of the origins of each question included in the questionnaire. [file 1471-2458-8-327-S10.doc]

**Questionnaire Version 41 Origin of Questions**

| **Appendix 10: ORIGIN OF QUESTIONS**  **Question numbers refer to version Mothers Questionnaire 38 3-09-2007**  The questionnaire combines three main points of origin for both categories included and for specific questions included in categories:   1. Some questions are included because they provide the information required for funded studies that have been incorporated into the baseline data collection for BiB. Specifically New Generis: NERC: Urban Regeneration. 2. Some sections replicate exisiting questionnaires or parts of questionnaires. 3. Some questions have been generated by the Born in Bradford team to reflect core areas of concern to the project,   Small changes have been introduced following piloting to reflect data collectors perceptions of acceptability and clarity in the question.  The following summarises sources for each questions and records changes made if the question came form a source other than the BiB team. | | | |
| --- | --- | --- | --- |
|  | **Title:** | **References** |  |
| **First Section** |  | Questions 1-7  Question 8.  Question 9 and 9a | From Born in Bradford Team* .  (*Throughout this form of words means discussed at Executive, Management or Scientific group and incorporating feedback from interviewers in the feasibility and pilot study )  From Professor Cameron – Loughborough (although precise wording is from the Born in Bradford Team.)  At request of Eamonn Sheridan and Yanick Crow – Genetics Dept BRI |
| **A** | Where you live | A1.  A2/A2a/A3 | Born in Bradford team  Health Survey for England 2004 – Household Questionnaire. |
| **B** | Who you live with | B1  B2.  B3) | Census 2001 – England Household Form – The Born in Bradford Team have modified this question to include the respondent in the calculation of numbers in the household. This change reflects feedback from the feasibility study.  Census 2001 – England Individual Form  From Census but modified by Born in Bradford Team: addition of “not living with a partner but in a relationship” comes from pilot study findings. |
| **C** | About you, your family and your baby’s father and his family. | C1/4/6/8  C2.  C3/5/7/9  C10 (all parts) | Country list is an abbreviated version of list on Census 2001 – England Household Form (The Born in Bradford Team have focussed on the countries most likely to be named by our population – as evident from feasibility study). Other countries can then be written in.  Arrived at by the Born in Bradford team  Information about provinces / districts of Mirpur and list of villages in Appendix A of notes for interviewers are from Azad Jammu and Kashmir Government Website [www.ajk.gov.pk](http://www.ajk.gov.pk/) (accessed 27/9/2006.) List of known names of Biraderi were supplied by Bradford Hospital and Leeds University genetics depts.  Health Survey for England 2004 – Household Questionnaire |
| **D** | Your family | D1, 2 and 3  D4 | These questions have been developed in consultation with Leeds Genetic Services, Bradford Hospitals Geneticists’ including Eamonn Sheridan, Dr Aamra Darr (University of Bradford) and by correspondence with Professor Alan Bittles (Perth, Australia).  Family tree – this has been developed by genetics departments in Leeds and Bradford Hospitals/ Leeds University. Adopted with advice of Leeds NHS Trust expertise on genetic mapping. |
| **E** | Education | E1/2  E1a/E2a  E1b//E2b | Census 2001 – England Individual Form.  Pakistan qualifications obtained from School of Lifelong Learning and Development, University of Bradford. These are standard classifications used re HEFCE admissions/credits procedures.  From the Born in Bradford Team |
| **F** | Current Employment | F1/F2/F3  F4/F5/F6/  F7/F8  F9/F10/F11  F12  F13 to F22 | From Born in Bradford Team.  Census 2001 – England Individual Form  From Born in Bradford Team.  These questions come from and relate to Mark Nieuwenhuijsen’s research study (Mark was originally at Imperial College, now at CREAL, Barcelona). There are designed to identify work and also transport to and from work as possible sites of environmental exposures.  Question is from Office of National Statistics – Standard occupational classification [www.statistics.gov.uk/methods - accessed 1/9/2006](http://www.statistics.gov.uk/methods - accessed 1/9/2006). But it has been modified to include further possible answers: Student: does not work. Also taxi-driver added into classification ‘Routine manual and service occupation).  Family Resources Survey – Maternal deprivation block Department of Work and Pensions Working Paper 28, 2006. Used after consultation with Professor J Bradshaw, University of York. |
| **G** | Smoking/Drugs/Alcohol | G1- to G4  G5  G6  G7 | **All** questions in Section G were influenced by the approach in the Health Survey for England 2004. The Health of Minority Ethnic Groups. The questions in the Health Survey have been modified following expert advice and the experience of the pilot phase of Born in Bradford.  Modifications draw on:  Questions from McKinney, Cade, Burley, Hepworth, Mijal (University of Leeds)  From Professor Bhopal (University of Edinburgh) – as used in previous studies and publications. The questions have been modified from those in Professor Bhopal’s papers following the pilot stage – a list of substances has been replaced with write in boxes. Interviewers have a prompt list of possible substances taken from Professor Bhopal’s work.  The B in B team have added cannabis/marijuana to the list following pilot phases where there was some evidence that people were prepared to provide information of illegal drug consumption.  Combination of suggestions from McKinney, Cade, Burley and Hepworth and Mijou in Leeds and Professor Leon (London). |
| **H** | Your diet | H1 to H 3 | From Janet Cade and her team and the New Generis Team.  McKinney, Cade, Burley, Hepworth, Mijal |
| **I** | Water consumption | I 1 to I 4 | Questions and justification from Imperial Team. Mark Nieuwenhuijsen’s NERC funded project and linked doctoral studies (Mark – originally at Imperial College, now at CREAL, Barcelona). |
| **J** | General Health (self-completed) | J 1 to J 4 | GHQ 28 Questionnaire |
| **K**  **L**  **M** | Exercise (self-completed)  Interviewers feedback  Your diet (self-completed) | K1 to K3  All questions in L  M1 to M3  M4 /M5 | General Practice Physical Activity Questionnaire (GPPAQ) October 2006  After piloting this was modified very slightly – reference to actual walking speeds was moved from self-completed section.  From Born in Bradford Team.  From University of Leeds: Janet Cade and her team and the New Generis Team. McKinney, Cade, Burley, Hepworth, Mijal  From Urban Regeneration (HEFCE) project – principal investigator Dr Nicola Lowe, University of Central Lancashire. |
