# Supplementary material for: Born in Bradford, a cohort study of babies born in Bradford, and their parents: Protocol for the recruitment phase
Source: BMC Public Health. 2008 Sep 23;8:327. doi: 10.1186/1471-2458-8-327 (PMC2562385; doi:10.1186/1471-2458-8-327)
Supplement: Additional file 11 — Interviewer notes. Notes provided to those administering the baseline questionnaire to standardise the way the questions are asked. [file 1471-2458-8-327-S11.doc]

| BORN IN BRADFORD BABY’S MOTHER **INTERVIEWER’S NOTES** |
| --- |

**Introductory Questions**

| Question 2.  Question 3.  Question 6 & 7  Question 8  Question 9  9a) | It is the language used for talking to the mother that should be recorded.  Put a cross in boxes to reflect all languages used eg – most of the interview may have been in English with just some questions in Mirpuri – so put a cross in English and Murpuri boxes.  Answer yes if an interpreter was used at all – even if they were only used for a short section of the questionnaire.  Sometimes triceps and arm circumference cannot be taken – for example when clothing is too tight and cannot be removed.  If not sure then prompt:-  was it a summer/winter  During school term/in holidays  Fill in the best estimate i.e. if they say spring and can’t be more precise assume this is:-  Spring 16 April  Summer 16 July  Autumn 16 October  Winter 16 January  This is to record births so all births are eligible even if the baby was still born or died subsequently.  If she has had more than five children continue to list in birth order in available boxes. If you run out of space just list on the page. |
| --- | --- |

**Section A - Where you live**

| A1.  A2  A2 and A2a) | If more than a year, write in number of years – record whichever is nearest to actual length of time ie if they have lived say two years and seven months round up to 3 years, two years and five months round down to 2 years.  If less than 1 year round up figures to the nearest month.    A household is:   - One person living alone or - A group of people (not necessarily related) living at the same address with common housekeeping – sharing either a living room or sitting room, or at least one meal a day   A ‘friend’ here means someone you were friends with before you moved to your present accommodation. |
| --- | --- |
| A3 | Exclude bedrooms converted to other uses (e.g. bathroom).  Include bedrooms temporarily used for other things  (e.g. study, playroom)  Even if bedrooms are not used count them.  If two rooms have been converted into one - count them as one room.  If one room has been converted into 2 - count as two rooms.  A bed-sit should be recorded as 1 bedroom. A one bedroom flat or house also as 1 |

**Section B – Who you live with?**

| B1 | Include – anyone who is temporarily away from home at present  - include schoolchildren or students who are away from home  during term time only.  - Include people with more than one address if they live at  your address for the majority of time.  - Include anyone who is staying with you who has no other  usual address.  - Include a husband or partner who works away from home, or is  in the armed forces, or is out of the country visiting relatives or working abroad (eg in Pakistan) and usually lives at your address. |
| --- | --- |

**Section C – About you, your family and your baby’s father and his family**

| C2  C3a)  Also applies to:  C5a)  C7a)  C9a)  C3b)  Also applies to C5b) C7b) C9b)  C4 and C6 C8  (may also apply to C1). | People sometimes move to a country for a short time then return home. Here you should note the age when the woman herself defines that she moved to the UK.  Map of Mirpur and list of towns and villages in ***Appendix A.***  This list may not be comprehensive, so please write down the town or village identified by the interviewee.  A Biraderi is a traditional social and / or occupational group. **Appendix B** lists biraderi names. This list may not be comprehensive, so please write down the town or village identified by the interviewee.  It may be that when asking about place of birth people born before 1950 may not have been born in Pakistan – that is Pakistan only came into existence as a country in 1947.  This question should be answered as if the current political boundaries had been in place at the time of their birth. This rule applies to all other countries eg Sri Lanka: Czech Republic: Slovakia: Bangladesh – all have ‘recent’ historical origins. It may also be that someone describes place of birth as Kashmir. This is in effect a political statement reflecting some reluctance to accept either Indian or Pakistani sovereignty over Kashmir. If Kashmir is given then it should be written into the Other box – but you should go on to fill in the next sections as if they had answered Pakistan ie after C1 go to C3 and so on. |
| --- | --- |

**Section D Your family**

| D1  D4 | See ***Appendix c***  for explanation about what a cousin is and various types of cousin. Also provided to the appendix is an example of a family tree to aid recall  If answered yes in D1 then a family tree should be completed on a separate form which includes the study number – please fill in question D4. |
| --- | --- |

**Section E Education**

| E1/E1A and E2/E2A | Cross highest qualification that applies.  If the person is not certain indicate don’t know. If they identify a qualification not on the list try and cross the box that is closest to what they have identified.  We are really interested in the level of their qualification – that is did they complete primary school only, primary and secondary only, secondary and further education only or higher education. |
| --- | --- |
| E1a) and E2a | See ***Appendix D***  for a list of Pakistani education qualifications.  The qualifications in E1a) and E2a) are those most often awarded in Pakistan. But they do change overtime and they also may be known under different names. If a qualification is identified that is not listed try and find what its nearest equivalent is and cross that box. |

**Section F Your current employment**

| F2  F4a | Employed means that you have a job – even if you are temporarily not working – see question F3  General type of company/industry e.g.,  Bank, Shop, Textile Factory. |
| --- | --- |
| F5b | We wish to record the number of people who work at the same place as our interviewee. They may work in a shop with one other person – in which case tick box 1-2 even if the shop is part of a chain of shops that employs hundreds of people.  Likewise if, say, they work at Tesco’s it is the number who work at the establishment they work in and not the overall number of people who work at Tesco’s in Bradford, or in the UK, or worldwide. This question is part of a national approach to classifying occupation and it helps us compare Bradford mothers with the national picture. |
| F6 | We want descriptions like:-   - cutting and sewing cloth - packing garments - writing in order book |
| F8 | In asking how long they have done a job we are trying to get at how long they have done the sorts of things they recorded in question F5c and F6 – that is how long they have for example been a solicitor rather than how long they have worked in a particular solicitors firm. This question is to give some background on how their job might impact on their health and well-being. |
| F9 | If person works in different places each day then indicate ‘no regular place of work’. E.g. They could be a door-to-door salesperson, a supply teacher, a carer. |
| F12  F13 F14/15/16 | If baby’s father lives away including out of the country – still seek an answer for this question. Appendix E provides examples of each type of job in the list.  There are cards provided with these questions – if it is easier you can show interviewees and ask them to indicate the correct answer. Then transfer that answer to the questionnaire booklet |
| F14  F15/1718/19/20 | We do not need to be exact – we are trying to get an idea of the level of income this individual/couple has – that is what’s important – the level rather than the exact amount.  These questions are somewhat culturally specific eg holiday away from home – doesn’t cover people who save for a few years to have an extended trip to relatives in Pakistan for example. If this is the case indicate “do not want/need this at the moment” : two pairs of all weather shoes – many women go through the winter with “summer” shoes as a choice – indicate “do not want/need this at the moment” |

**Section G Smoking/Alcohol/Drug Use**

| G1 to G4  G5  G7a)  G7b)  G7e)  G7h)  G7i) | Just relates to cigarettes and cigarette smoke  See card with list of possible products for this section  Occasionally means less than once a week eg just on special occasions  List of units of alcohol in typical drinks  A pint of ordinary strength lager (Carling Black Label, Fosters) – 2units  A pint of strong lager (Stella Artois, Kronenbourg 1664) – 3 units  A pint of ordinary bitter (John Smith’s, Boddingtons) – 2 units  A pint of best bitter (Fuller’s ESB, Young’s Special) – 3 units  A pint of ordinary strength cider (Woodpecker) – 2 units  A pint of strong cider (Dry Blackthorn, Strongbow) – 3 units  A 175ml glass of red or white wine – around 2 units  A pub measure of spirits – 1 unit  An alcopop (eg Smirnoff Ice, Bacardi Breezer, WKD, Reef) – around 1.5 units.  (Source: Department of Health Website)  <http://www.dh.gov.uk/en/PolicyAndGuidance/HealthAndSocialCareTopics/AlcoholMisuse/AlcoholMisuseGeneralInformation/DH_4062199> |
| --- | --- |

**Section H Your Diet.**

**H2** If don’t know if caffeinated or not then count as caffeinated

**Section I Water Consumption**

| I1 | If you boil water before drinking it record this as tap water.  One glass is 200ml , one cup is 200ml (200ml is about the amount in a full wine glass)  One mug is equal to two cups |
| --- | --- |
| I2 and I 3 | A water filter or filter jug – if they are unsure what these are then indicate don’t know |

**Section J General Health**

| Section J | This section should be completed by the person themselves.  Please sit with them while they complete these sections and answer any queries.  While you are waiting you can check the other parts of the questionnaire to check everything has been completed.  Questions that have caused some confusion include J1b) here ‘a good tonic’ means feeling a need of something that would make them feel more energetic.  Please note: If interviewees find section J distressing then tell them they can seek the support of: |
| --- | --- |

**Section K – Exercise**

**Section k (self-completed)**

**K3** walking pace is as defined by the interviewee ie what they think is slow or fast.

**After Section J and K Interviewee to pass the questionnaire back to Interviewer.**

| If people want to make comments about the interview please given them the chance. |
| --- |

**Section M Your Diet.**

This section is completed while the mother is in the GTT waiting area. It is supposed to be self-explanatory. If you are asked any questions the following might help: M1/ 2/ 3 – if they are not sure if a food they eat fits into a particular category get them to make there best guess

M3 “well done” is also according to their own estimate.

**Section Interviewer’s feedback**

| This is for you to complete.  L4 is to see if you felt they were providing truthful/reliable comments. We want your subjective impressions.  When you have finished all sections and checked them yourself please get another member of the team to check the completed questionnaire to see all sections have been completed – including the self-completed sections. They should then add their name/number |
| --- |
