# Supplementary material for: Born in Bradford, a cohort study of babies born in Bradford, and their parents: Protocol for the recruitment phase
Source: BMC Public Health. 2008 Sep 23;8:327. doi: 10.1186/1471-2458-8-327 (PMC2562385; doi:10.1186/1471-2458-8-327)
Supplement: Additional file 12 — Quality control standards for growth measurements. Quality monitoring standards for collection of anthropometric measurements. [file 1471-2458-8-327-S12.doc]

**Quality Control Standards for Growth Measurements**

The reliability of data collected is of critical importance when deciding on which measurements to take and what technique to use. A lack of reliability can make collection of anthropometric measurements (growth data) meaningless.

There are three sources of error:-

1. The natural variation of the measurement itself:

- height varies during the course of the day because of postural changes

2. Instrument error:

- a poor quality equipment will provide incorrect readings e.g.(poorly calibrated weighing scale)

3. The observer:

- poor or inadequate training
- no practice will cause a measurer to alter their technique
- significant differences between different measurers.

All of these errors can be reduced by frequent checks on equipment, proper training, and a commitment by the measurer to ensure that they measure consistently. These quality monitoring procedures will help to reduce variation over time (day-to-day, week-to-week) by the same single measurer and between measurers. However, with a longitudinal study, such as Born in Bradford (BiB), continual checks need to be in place to check the degree of reliability of a given measurement*.*

**How do we make sure our measurements are reliable over time?**

We use the **Technical Error of Measurement** (TEM[[1]](#footnote-2)) which can provide sufficient information to determine whether a set of anthropometric measurements should be deemed reliable.

A TEM is calculated from the data produced by a **test-retest** reliability study. As the title suggests a test-retest study requires the measurer to assess the same infant twice (intra-observer reliability) or two or more observers to assess the same infant (inter-observer reliability).

Born in Bradford needs to calculate the TEM at the beginning of the study and then monitor the quality of data over time by repeat test-retest studies. However it obviously takes some time to get the initial reliability studies so until we have sufficient data we have suggested acceptable ranges of difference between two measurements on the same subject.

**Acceptable Ranges for repeat measurements**

| **Measurement** | **BIB**  **Acceptable Range** |
| --- | --- |
| Weight (kg) | 0.2 |
| Length (cm) | 1.0 |
| Head Circ (cm) | 0.5 |
| Abdominal Circ (cm) | 1.0 |

**Justification for BiB figures**

The *“BiB* acceptable range” figures are roughly double the Frisancho ^1^ figures to give an initial target to aim for when training, and before BiB has produced its own TEMs.

**Test-retest reliability studies**

Reliability studies should be carried out on a three to six monthly basis. Born in Bradford Trailblazing Teams will pilot the feasibility of six-weekly or bi-monthly “Reliability weeks” (RW). The Born in Bradford Research Health Visitor and Will Johnson will visit the measuring sites to check on procedures and provide help and guidance where necessary.

**Intra-observer:** Each measurer will be required to measure the same subjects twice. The important data are the differences between the measurements on occasion 1 and occasion 2.

We suggest that the first 5 to 10 subjects on a designated “reliability week” be the experimental group. Ideally the repeat set of measurement will not follow directly after the first set but there will be a time-lag of at least 10 minutes.

**Inter-observer:** Two (or more) measurers need to measure the same subjects once each. The important data are the differences between the measurements of observer 1 and observer 2.

If the reliability studies are organised such that HVs work in pairs and that partnerships change regularly then overall reliability should be enhanced i.e. familiarity will not breed lowered reliability.

Data will be analysed centrally (by Will Johnson) and the results fed back directly to the BiB Trailblazing HV teams. The BiB Research Health Visitor and Will Johnson will visit these teams on a regular basis to monitor reliability studies and quality control.

REFERENCES:

1. Frisancho RA. *Anthropometric standards for the assessment of growth and nutritional status.* Ann Arbor: The University of Michigan Press; 1990.

1. Technical note: the TEM is the standard deviation of the differences between repeat measurements i.e. SQRT. Σ d^2^_12_ / 2N [↑](#footnote-ref-2)
