# Supplementary material for: Born in Bradford, a cohort study of babies born in Bradford, and their parents: Protocol for the recruitment phase
Source: BMC Public Health. 2008 Sep 23;8:327. doi: 10.1186/1471-2458-8-327 (PMC2562385; doi:10.1186/1471-2458-8-327)
Supplement: Additional file 13 — Table 1. Acceptable ranges of difference between the two measurements on the same person. [file 1471-2458-8-327-S13.doc]

**Table** 1 Acceptable ranges of difference between two measurements on the same person

| **Measurement** | **Acceptable range** |
| --- | --- |
| Weight | 200 gm |
| Length | 1 cm |
| Abdominal circumference | 1 cm |
| Head circumference | 5 mm |
| Triceps | 2 mm |
| Subscapular | 1 mm |
