# Supplementary material for: Born in Bradford, a cohort study of babies born in Bradford, and their parents: Protocol for the recruitment phase
Source: BMC Public Health. 2008 Sep 23;8:327. doi: 10.1186/1471-2458-8-327 (PMC2562385; doi:10.1186/1471-2458-8-327)
Supplement: Additional file 14 — Advocacy and scrutiny committee. Terms of reference for the Advocacy and Scrutiny Committee. [file 1471-2458-8-327-S14.doc]

**ADVOCACY AND SCRUTINY COMMITTEE (ASC) - 12/03/07**

**Term of Reference and views on the committee’s community responsibilities of the Born in Bradford (BiB) Project**

1.Introduction

The A.S.C. has been set up as an independent review group to support and advocate on behalf of parents recruited into the Born in Bradford Study. The committee will form part of the formal structure of the BIB project and will work alongside the Executive Committee for the project (see flowchart) and develop a clear process and relationship with the Scientific Committee.

The committee wish to operate in an interactive and dynamic way with the wider public and enrolled parents rather than a traditional committee format and develop and explore new ways of engagement with the project. This will mean working with community groups,and specific bodies or health groups from a wide range of public arenas.There will also be a role for associate members who will be individual parents or professionals who may be called upon to assist the work of the A.S.C. in its role at different stages of its work for a specific period of time.

It is anticipated that the committee will comprise of two co- chairs,one bringing a parents perspective and the other a professional one ,there will be six professional members, six parent members and two observers from the project who will have no voting rights on the committee but will administrate the committee and have responsibility for updating the members on the project developments.

There will be a review of the membership in July and a further review in twelve months time at which point the terms of office for membership and Chair will be agreed. The Terms of Reference will also be revisited.

Initially the committee will work with the Executive Group to establish the flow of information to the A.S.C. and its relationship with the Scientific Committee within the Terms of Reference .

**TERMS OF REFERENCE**

1. To set standards of best practice in terms of communication,consultation and

feedback to parents and the public.

2. To act as advocates for parents,carers,and children,protecting their interests and ensuring relevant policies are in place.

3. To receive and initiate action on any concerns or complaints received from parents,carers and children in the study.

1. To act as an independent advisory body for the project in terms of

- - all new and nested studies
  - new questions
  - participant information
  - recruitment
  - parental feedback

**The Advocacy and Scrutiny Committee’s views on the community responsibilities of the Born in Bradford (BiB) Project**

As members of the Advocacy and Scrutiny Committee (ASC), with both professional and non-professional interest in supporting the work of the Born in Bradford study, the ASC would like to emphasise the importance of sensitive presentation of future research findings both by the BiB staff and by any researchers using BiB data and biobank materials for their own research purposes.

The people being recruited to the project are told that this is a health study based in Bradford for the better health of the people of Bradford. Bradfordians give their consent willingly in their generous wish to help to identify the causes of the major childhood illnesses and to prevent them happening to future generations. BiB has the goodwill of the people it has recruited and of many other supporters.

It is important for the BiB project’s relationship with the people of the City that it is seen to be open and honest with its research findings. The ASC feels that BiB should echo the aims of the ‘Bradford Commission Report in to the Bradford Riots of 1995' in a wish to promote ’peace, harmony and understanding between the communities of Bradford’ in the presentation of any research outcomes.

Bradford has a diverse multilingual and multicultural population and it is with this in mind that the ASC supports BiB commitment to presenting their research findings in ways that can be understood by most people. Efforts should be made to explain the research in many ways and in many settings so that all communities are included.

BiB should be ready to liaise with other health providers in the city to have support and guidance ready for those affected by the findings.
