# Supplementary material for: Born in Bradford, a cohort study of babies born in Bradford, and their parents: Protocol for the recruitment phase
Source: BMC Public Health. 2008 Sep 23;8:327. doi: 10.1186/1471-2458-8-327 (PMC2562385; doi:10.1186/1471-2458-8-327)
Supplement: Additional file 15 — Guidance and conditions for collaborators on the Born in Bradford programme. This document outlines the procedures put in place to deal with applications to conduct nested studies; requests to access and use study data and publication and intellectual property protocols. [file 1471-2458-8-327-S15.doc]

**Guidance and conditions for collaborators on the Born in Bradford programme: new proposals, use of data, publication and intellectual property**

Born in Bradford is a platform study that aims to increase our understanding about how we can improve health and well-being in the City. It also aims to contribute to international knowledge and understanding about causes of disease and promotion of health. The study was also set up to increase research capacity in Bradford, to attract high calibre research partners and grow local research talent. The study intends to open up opportunities for career development for local researchers and increased access to academic support.

The implementation of the study is led by NHS partners, Bradford Teaching Hospitals NHS Foundation Trust and the Bradford and Airedale PCT, and is dependent on the commitment and contributions of a wide variety of Bradford health professionals. These include midwives, obstetricians, paediatricians, health visitors, health service managers and general practitioners. Academically it has been led by the Universities of Bradford and Leeds, but with major contributions from researchers in the Universities of Bristol, Loughborough, Edinburgh and London. The most important partners of all are the parents and children recruited into the study.

Born in Bradford is therefore dependent on the input and contributions of a large number of stakeholders. This plurality is a unique strength of the study, but may also lead to confusion over ownership. In addition to the platform study there are likely to a significant number of nested studies. This document confirms the relationship between these and the platform study. Specifically, the following principles have been proposed to clarify intellectual property and provide guidance for authorship and grant applications.

**New proposals**

1. Researchers with proposals for the project should in the first instance complete an outline proforma available on the Born in Bradford website ([www.borninbradford.nhs.uk](http://www.borninbradford.nhs.uk/)) and submit to the Director (Dr John Wright). These will be reviewed by the Executive group and if approved a more detailed proposal will be invited. Approved studies will be reviewed by the Scientific Collaborators group. Proposals for use of biological specimens will require approval by the Biobank Access Committee.
2. All grant applications and academic papers should ensure that there is a local (Bradford) investigator. A key aim of the study is to develop research capacity in Bradford. Research collaborators should ensure that there is genuine local research partnership.
3. The costs of administrative, data management or laboratory processing incurred by the BiB programme in providing the data will be reimbursed by the applicants.
4. The Advocacy and Scrutiny Committee will consider all research involving the Born in Bradford cohort at an early stage to contribute to local acceptability and appropriateness.
5. The Born in Bradford Executive Group will act as data guardians to provide peer review and approve the scientific merit of research ideas and the use of the collected data and biological samples.

**New data or derived variables**

1. Any new data derived from either biological samples or existing variables must be lodged with the main database at the end of the project date (or at any time at the request of the BiB Director). The nested study Principal Investigator must supply adequate documentation concerning new variables (including statistical programs) to permit their use in future analyses of the data.
2. No other investigator will have access to any new data produced by the collaborator without their prior knowledge and an opportunity to comment on its appropriateness. It is assumed that under usual circumstances the applicants will have an active involvement in any future work that uses their derived variables. The BiB Director will adjudicate any irreconcilable differences between investigators.
3. Any residues of biological samples or excess materials must be returned to the BIB Biobank by the end date of the project. The expense of transferring both from and back to the BiB site must be met by the applicants.
4. The applicants must notify the BiB Director of any potential errors discovered with using the materials.

**Publications and reports**

1. All research outputs from the study should clearly acknowledge the contribution from the Bradford community. The cohort study has been set up to attempt genuine public engagement and the Born in Bradford cohort should therefore been seen as active contributors rather than just passive participants.
2. The standard acknowledgement for all publications will include the following:

*We are grateful to all the families who took part in this study, the midwives for their help in recruiting them, paediatricians, health visitors, the Born in Bradford team which included interviewers, data managers, laboratory staff, clerical workers, research scientists, volunteers and managers.*

1. Authorship credit should be based on accepted practice that there should be substantial contribution to the conception and design of the study, or analysis and interpretation of data, and drafting the article or revising it critically for important intellectual content. Authorship will be agreed at the start of any new research project to avoid later disputes.
2. When a paper is ready to be submitted for publication, but prior to its submission, the lead author will be required to submit a copy (in confidence) to the Executive Group. We expect to process all papers within two weeks of receipt. We read all papers to check confidentiality is protected; to ensure that the paper will not bring the study into disrepute; to try to identify overlap with other papers published or in preparation. We also provide advice and feedback to authors where we feel this may be helpful.
3. When a paper is accepted for publication the lead author will be required to submit a copy to the Advocacy and Scrutiny Committee prior to publication. A checklist for authors provides guidance.
4. If no publications have been submitted by one year after the end date of a nested project, or no acceptable reasons for this absence of submission, or where there is doubt concerning the quality of the work, the BiB Executive reserves the right to ask other teams to investigate the same issue.

**Governance and intellectual property**

1. The Director for Born in Bradford will be responsible for the design and conduct of the platform study, ethical approval and compliance with research governance requirements.
2. Bradford Teaching Hospitals Foundation Trust is the Sponsor of the project.
3. Background Intellectual Property used in connection with studies is and will remain the property of the Bradford Institute for Health Research. Collaborators shall own any Intellectual Property Rights generated as part of their own research.

.
